# Population affinity and variation of sexual dimorphism in three-dimensional facial forms: comparisons between Turkish and Japanese populations

**DOI:** 10.1038/s41598-021-96029-9

**Published:** 2021-08-17

**Authors:** Chihiro Tanikawa, M. Okan Akcam, Hatice Gokalp, Edlira Zere, Kenji Takada

**Affiliations:** 1 Department of Orthodontics and Dentofacial Orthopedics, Graduate School of Dentistry, Osaka University, Suita, Osaka Japan; 2 Center for Advanced Medical Engineering and Informatics, Osaka University, Suita, Osaka Japan; 3 Department of Orthodontics, School of Dentistry, Ankara University, Besevler, Ankara Turkey

**Keywords:** Dental anthropology, Craniofacial orthodontics, Anthropology, Three-dimensional imaging

## Abstract

Examining the extent to which sex differences in three-dimensional (3D) facial soft tissue configurations are similar across diverse populations could suggest the source of the indirect evolutionary benefits of facial sexual dimorphism traits. To explore this idea, we selected two geographically distinct populations. Three-dimensional model faces were derived from 272 Turkish and Japanese men and women; their facial morphologies were evaluated using landmark and surface-based analyses. We found four common facial features related to sexual dimorphism. Both Turkish and Japanese females had a shorter lower face height, a flatter forehead, greater sagittal cheek protrusion in the infraorbital region but less prominence of the cheek in the parotid-masseteric region, and an antero-posteriorly smaller nose when compared with their male counterparts. The results indicated the possible phylogenetic contribution of the masticatory organ function and morphogenesis on sexual dimorphism of the human face in addition to previously reported biological and psychological characteristics, including sexual maturity, reproductive potential, mating success, general health, immune response, age, and personality.

## Introduction

Human facial sexual dimorphism is considered to be associated with a multidimensional nature and general significance, including sexual maturity, attractiveness, reproductive potential, mating success, general health, immune response, age, and personality^1–7^. Examining the extent to which sex differences in facial soft tissue configurations are similar across diverse populations may suggest the source of the indirect evolutionary benefits of facial sexual dimorphism traits.

With modern data related to different human populations, sexual dimorphism of craniofacial hard tissue was previously analyzed in several researches to explore human evolution^8^. This was because craniofacial hard tissue was considered to reflect population history and sexual dimorphism to varying degrees. A study of the zygomatic bone shape was extracted from the computer tomography data of 98 Chinese and 96 Germans; it was found that population-related shape differences were captured primarily and sexual dimorphism were less distinct compared with the population differences^9^. Another study^10^ examined a sample of modern human crania (n = 281) designed to represent modern human geographic variations, as well as diverse subsistence activities, that can be subdivided into 14 main regional/genetic groups; they reported that robusticity of the cranial shape between males and females, was consistently different in different populations. Other researchers^11^ also studied cranial traits among different populations of modern African American, European American, and English groups; they observed greater variations in the sexual dimorphism of cranial traits. A separate study that evaluated the shape of the chin, among specimens of nine geographic regions, showed that sex differences were not geographically universal^12^. Consequently, population affinity in sexual dimorphism of hard tissue remains controversial.

Sexual dimorphism of craniofacial soft tissue has also been studied in two dimensions and three dimensions. Recent evidence on cross-populational variation in the patterns of (and preferences for) facial sexual dimorphism based on 1307 individuals from 8 distinct human populations using two-dimensional (2D) photographs showed that facial dimorphism differs substantially across human populations^13^. The study further showed that facial sexual dimorphism is low in Africa but high in South America and Europe. Furthermore, their analysis distinguished between allometric and non-allometric facial components, revealing significant differences in facial dimorphism between populations due to allometry. Interestingly, there is still some consistency in the direction of morphological changes associated with dimorphism.

A previous study compared three-dimensional (3D) facial morphologies in two European Caucasian populations from the UK and Netherlands, and found that females had greater human variability than males, which indicates non-universal sexual dimorphism^14^. A more recent study of 3D facial photos on two cross-sectional cohorts of children from Tanzania and the United States showed, through principal component analysis (PCA) and multifactor analysis of variance (MANOVA), that both allometric trajectories and sexual dimorphism were detectable, but were actually very similar in the two populations^15^. Although these studies described overall variation between the analyzed samples, detailed common sexually dimorphic characteristics among populations have not yet been described in 3D. Few studies have examined common 3D facial characteristics among populations to discuss the biological importance of oral and facial functions (including masticatory functions) in the evolution of each facial characteristics.

Although an individual’s ability to intake proper amounts of food with a healthy chewing apparatus is a prerequisite for natural selection in both sexes, men require more calories than women to sustain a healthy weight^16^ among different populations. The greater maximum molar bite force and masticatory muscle thickness of men^17^ may be related to their need for more calories compared with the corresponding need of women, Additionally, masticatory muscle size has a major impact on cranial structure^18^. Therefore, from a phylogenetic perspective, it is reasonable to assume that a well-developed masticatory apparatus in males is associated with the expression of facial sexual dimorphism with respect to both size and shape even among different populations. However, our understanding of this subject has been limited, because few studies previously investigated the impact of the masticatory apparatus and muscle function on three-dimensional facial sexual dimorphism in Homo sapiens.

Several researchers have discussed the hominid evolution and diets^8,19^. In order to resist the stress caused by frequently chewing tough foods, species that eat tougher food tend to have a thicker lower body of the jaw and mandibular joint^8^. The mandibular symphysis is placed relatively higher in order to increase the momentum of the masticatory muscles or to perform a more uniform occlusion in the entire cheek teeth^19^.

In this study, we examined the presence of population affinity in sexual dimorphism that may help elucidate the importance of the masticatory organs based on 3D facial surface configurations. Comparing sex differences in facial soft tissue configurations among different populations can provide insight into the evolutionary origin of facial sexual dimorphism. Briefly, because common characteristics between geographically distinct populations are thought to result from common evolutionary forces across populations, detecting possible relationships between these facial characteristics and the masticatory apparatus could strengthen our understanding of the importance of the masticatory apparatus or muscle function in the evolution of facial sexual dimorphism. Therefore, we analyzed sexual dimorphism in Turkish and Japanese populations, which are geographically distinct and have a diverse European/Middle-East population and homogenous Far-East population, respectively^20^.

In our study^21^, we determined a new set of variables that were effective for characterizing the differences in 3D facial surface configurations between males and females in the Japanese population. The study revealed site specificity and strength or intensity of sexual dimorphism with regard to facial soft tissue. A 3D facial analysis may contribute to a deeper analysis of the face, which may enable us to discuss the source of the indirect evolutionary benefits of facial sexual dimorphism traits in detail. The hypothesis of our research is that there is population affinity in sexual dimorphic faces and its common characteristics can be explained by the masticatory functions.

The objectives of the present study were:

1. To investigate the sex differences in 3D facial soft tissue configurations that are congruent in both Japanese and Turkish populations and those that are specific to each population, and thus to determine the sex-specific variation between and within populations.
2. To infer possible reasons that account for the observed commonality and dissimilarity from a biological aspect, especially with regard to the phylogenetic importance of the chewing apparatus.

## Results

### Averaged faces and accentuated averaged faces

**Figure 1:**
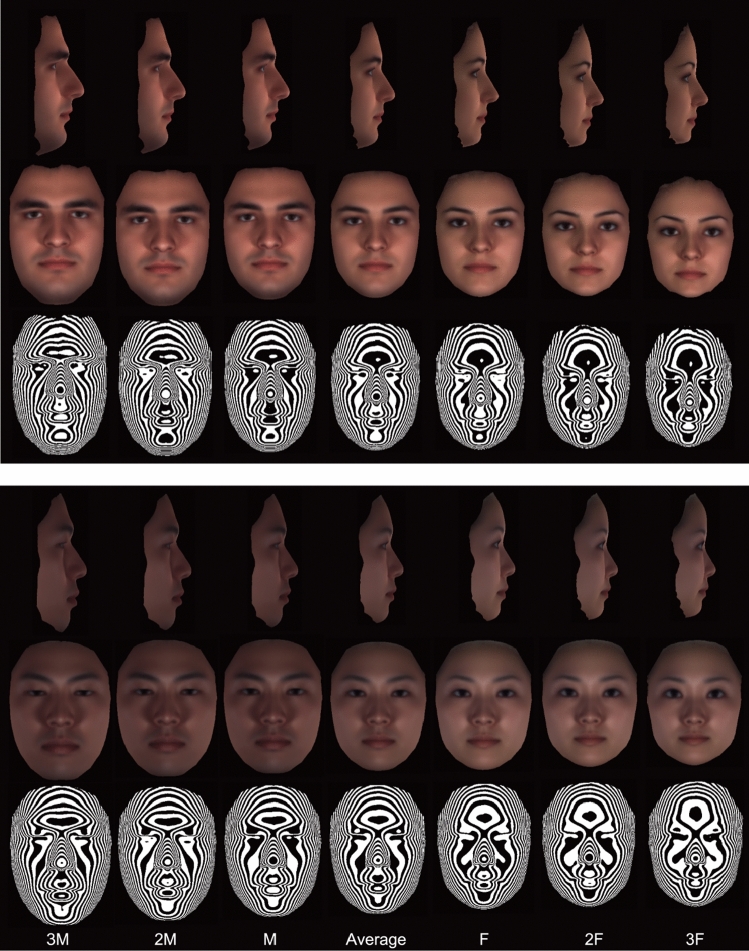
Computer-generated modeled faces that enhance understanding of sexual dimorphism in young human adults by demonstrating site-specific accentuated facial topography in Turkish (top) and in Japanese (bottom) populations. M and F, averaged male and female faces, respectively. Average, the averaged face of all subjects; 2 M and 3 M, the accentuated averaged male face weighted by 2 and 3, respectively (i.e., *w* = 2, 3); 2F and 3F, the accentuated female face weighted by 2 and 3, respectively (i.e., *w* = 2, 3). Top, lateral view; middle, frontal view; bottom, contour map. A commercial software (HBM-Rugle, Medic Engineering Co., Kyoto, http://www.rugle.co.jp/) was used to create this figure.

Figure 1 depicts the averaged and accentuated averaged faces that were computed for the male and female Turkish and Japanese subjects. The faces with enhanced masculinity and femininity that were mathematically calculated based on the data employed in this study clearly revealed that female faces differed from male faces in 3D. In general, facial differences associated with sex in both population groups were similar: females had a shorter face height, especially in the lower third of the face; a flatter forehead; and more rounded cheeks with a greater naso-labial fold.

### Surface-based analyses

**Figure 2:**
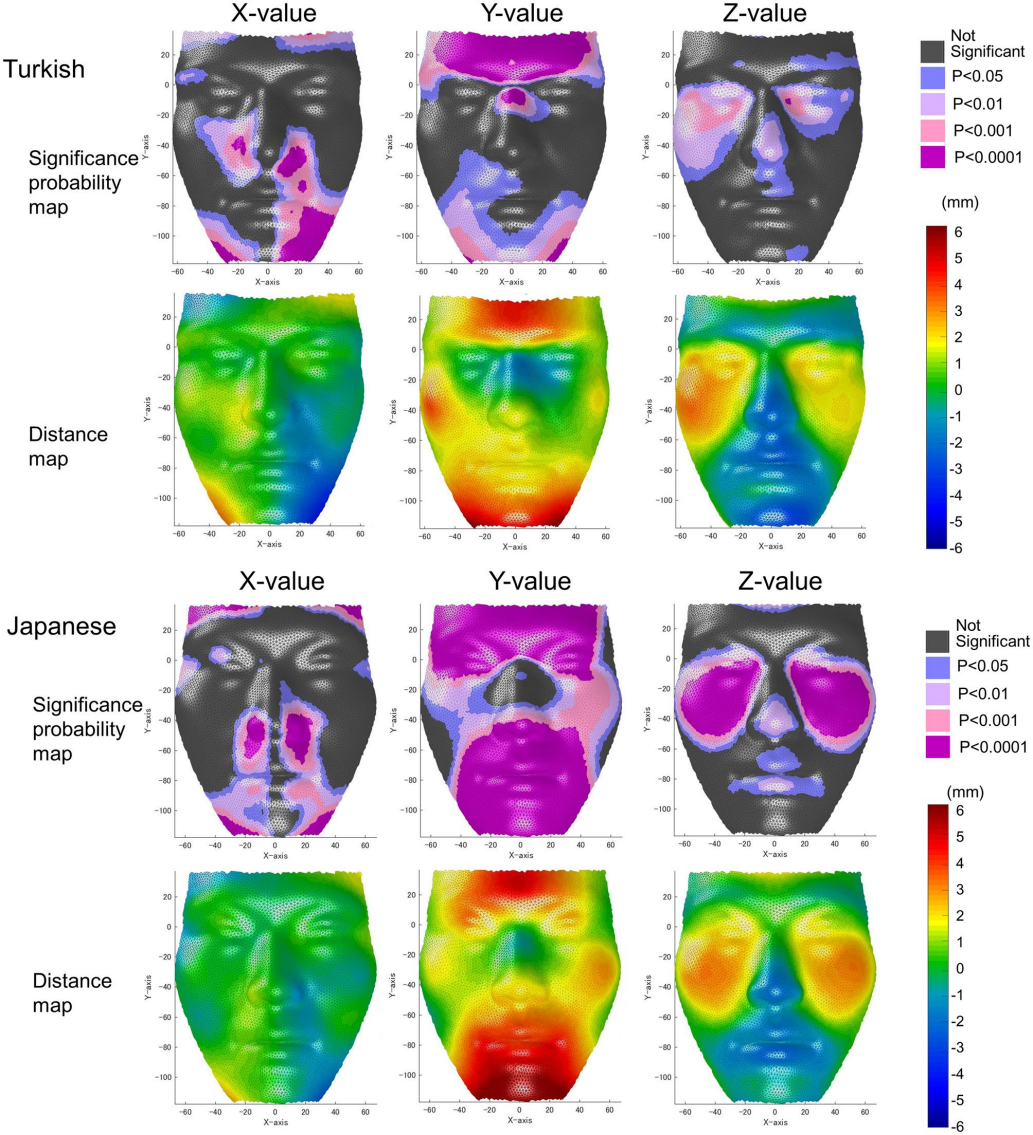
Significance probability maps [top (Turkish) and second from the bottom (Japanese)] and difference maps [female minus male; second from the top (Turkish) and bottom (Japanese)]. For the significance probability maps, blue designates P ≤ 0.05; pale pink, P ≤ 0.01; dark pink, P ≤ 0.001; and purple, P ≤ 0.0001. For the difference maps, red indicates that the female group exhibited greater values than the male group, whereas blue indicates that the male group exhibited greater values than the female group. Differences are represented in mm. A custom-made MATLAB based software (MATLAB 2021a, The MathWorks, Inc., Natick, MA) was used to create this figure.

Figure 2 shows significance probability maps and actual differences of the X-, Y-, and Z-values, and Supplementary Fig S1 represents mean vectors from male to female subgroups in each group.

#### X-value (transverse direction)

The most significant differences in the x-axis were observed in the nasal alar and chin in both population groups. Both population groups showed a smaller nasal alar width and smaller chin width when normalized by the distance between right and left exocanthions (P < 0.05). In the Turkish group, there were differences with respect to the left corner of the mouth, but not the right corner, on the X-axis; this meant that Turkish males had more laterally positioned corner of the mouth on the left side than the Turkish females.

#### Y-value (vertical direction)

The significance probability maps clearly showed that differences associated with sex were most prominent in the Y-axis among the three dimensions, especially in the Japanese group. The areas that showed significant differences were widely distributed along the lower faces; remarkable upward displacement was observed for the female subgroups, and this was consistent in both population groups. This characteristic led to an apparent decrease in the vertical height of the lower facial height in the female subgroups.

Furthermore, the supraorbital ridges also showed downward displacement in the male compared with female subgroups of both population groups, which indicates that the male subjects had greatly sloped supraorbital ridges and female subjects had flatter foreheads in both population groups.

In contrast, upward displacement of the subnasal region and the nasal tip in the female compared with male subgroup was only observed in the Japanese group; this trait is a sexually dimorphic phenotypic characteristic that was unique to the Japanese subjects. Moreover, greater upward displacement of the cheeks in the female subgroup was also only observed in the Japanese subjects.

The nasal dorsum showed greater upward displacement in the Turkish male subgroup, which indicates a greater naso-frontal angle and greater nasal hump (P < 0.05); this characteristic was unique to the Turkish subjects.

#### Z-axis (antero-posterior direction)

Z-value comparison showed the most consistent results between the two population groups. Both population groups had more protuberant cheeks in the female than in the male subjects. Additionally, females of both population groups had more retruded subnasal regions than males, and males of both population groups had significantly more protuberant nasal tips than females. The upper and lower lips were more retruded in the Japanese female group compared with the Japanese male group (P < 0.05), and this characteristic was unique to the Japanese population.

### Variance of each population and each sex, and their interactions

**Figure 3:**
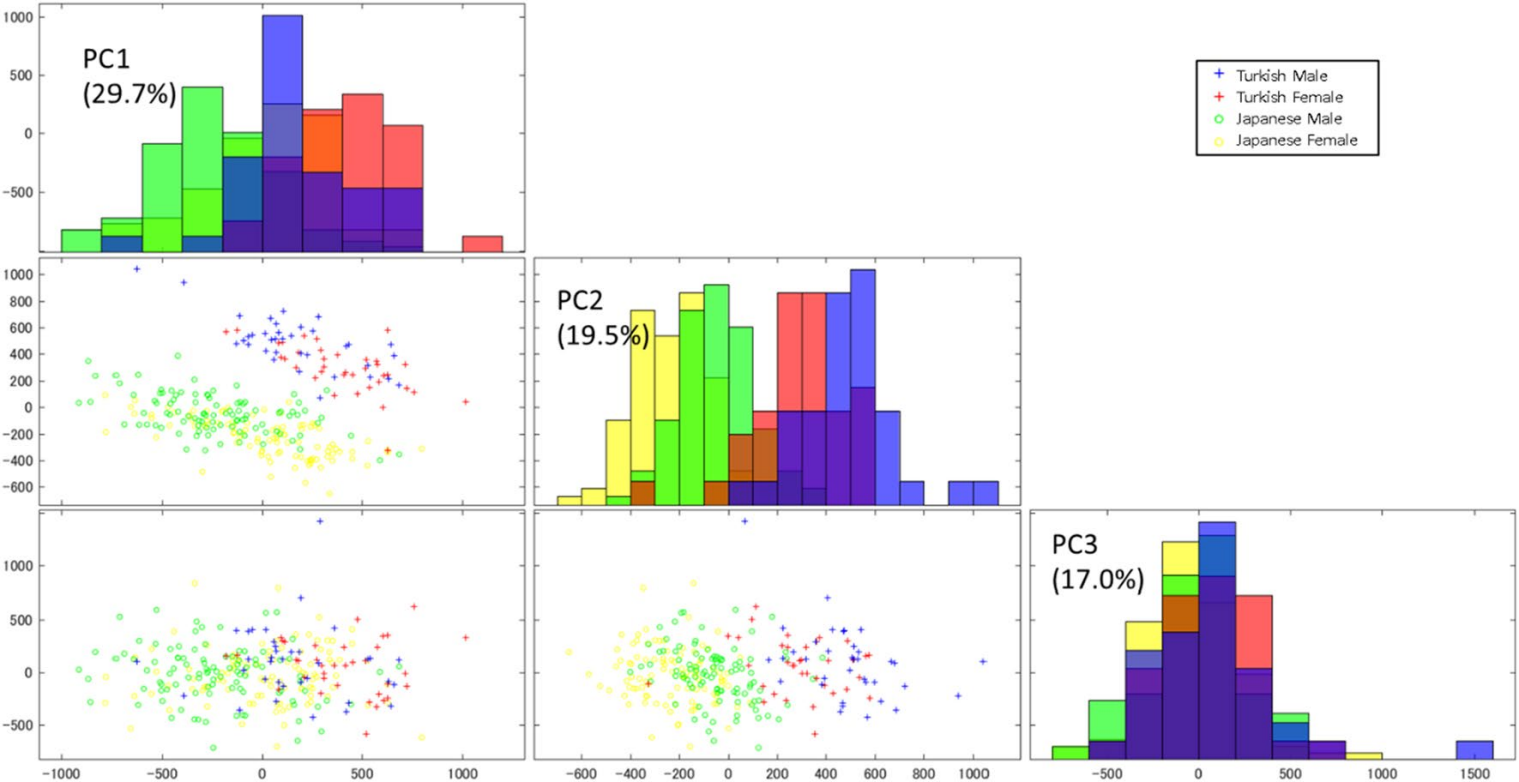
A scatter plot matrix of the principal component (PC) scores for Turkish and Japanese males and females with a histogram in diagonal cells. The second PC shows a clear separation between populations. In PC 1, yellow (Japanese females) is not visible as it is fully overlapped by green (Japanese males). Shape changes associated with PCs 1–3 are shown in Fig. 4.

**Figure 4:**
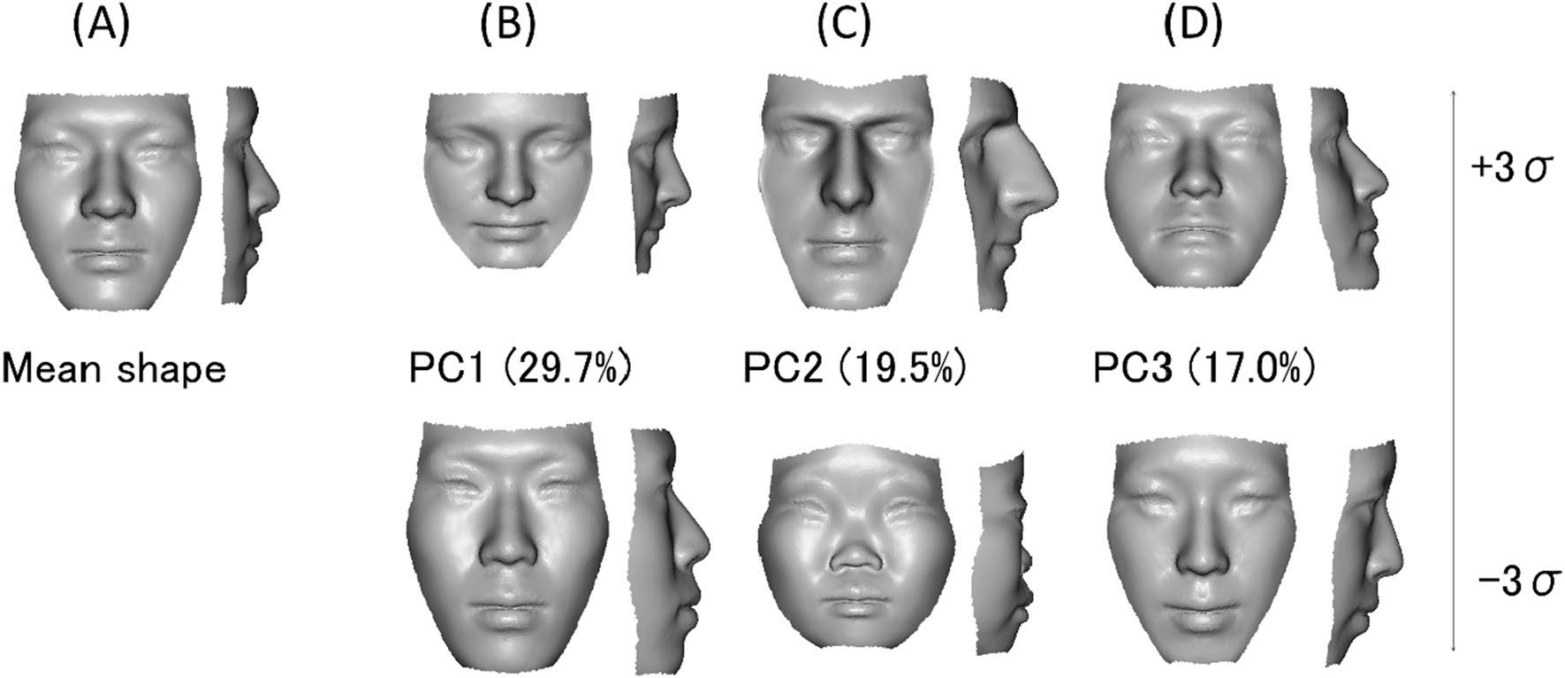
Principal components analysis (PCA) results. PCs 1–3 explains 66.2% of shape variation across samples. (**A**) Mean shape; (**B**) Shape changes associated with PC1, which explains 29.7% of shape variation across samples; (**C**) Shape changes associated with PC2, which explains 19.5% of shape variation across samples. (**D**) Shape changes associated with PC3, which explains 17.0% of shape variation across samples. A custom-made MATLAB based software (MATLAB 2021a, The MathWorks, Inc., Natick, MA) was used to create this figure.

The first three principal components (PCs), which explained 66.2% of the sample’s variance, were determined to be significant by a scree plot analysis. As the first three eigenvalues of a population covariance matrix were large enough compared to the others, PCA was considered to be appropriate for dimensional reduction of the present high dimensional data that exceeds the observation numbers^23^. Visualization of the between-group structure of the surface data (Figs. 3, 4) revealed a distinct separation between populations and, to a lesser extent, a noticeable expression of sexual dimorphism. These differences in population and sex were explained mainly in PCs 1 and 2. The shape variation of PC 1 was related to the size of the anterior lower facial height (Fig. 4); PC 2 was associated with either dolichocephalic or brachycephalic characteristics (with a positive value indicating an anterior-posteriorly greater head depth relative to its width with the protruded nose and chin); PC 3 was related to facial divergence (with a positive value associated with anterior divergence). Mahalanobis distances between the two population groups were 32.2 and 30.4 for the male and female subgroups, respectively. In contrast, Mahalanobis distances between the sex subgroups were 1.4 and 1.6 in the Japanese and Turkish groups, respectively (Supplementary Fig S2). These results indicate that the geographic variation in facial morphology was greater relative to within population variation related to sex. Sex and population affinity were highly significant P < 0.01, Table 1), but there was no significant interaction of these two factors (P = 0.88). This suggests that sexual dimorphism in facial configurations (explained by at least 66.2% of the sample’s variance) is consistent, regardless of the population.

**Table 1:** Multifactor analysis of variance (MANOVA) of the surface-based model.

	Df	Pillai	Eta^2^	Approx F	Num Df	Den Df	Pr(> F)
Population	1	0.30	0.17	36.65	3	262	< 2e−16
Sex	1	0.73	0.53	231.53	3	262	< 2e−16
Population: sex	1	0.00	0.00	0.23	3	262	0.88
Residuals	264						

### Allometric and non-allometric components of sexual shape dimorphism (SShD)

Decomposition of SShD into allometric and non-allometric components is shown in Supplementary Fig. S3 as a violin plot. The permutation test showed no significant differences between the Turkish and Japanese populations in the measures of the centroid size (CS), SShD, allometric SShD, and non-allometric SShD (p = 0.66, 0.97, 0.48, 0.21, respectively). This suggests that sexual dimorphism and its allometric and non-allometric components in facial configuration (again, explained by at least 66.2% of the sample’s variance) were consistent, regardless of the population.

**Figure 5:**
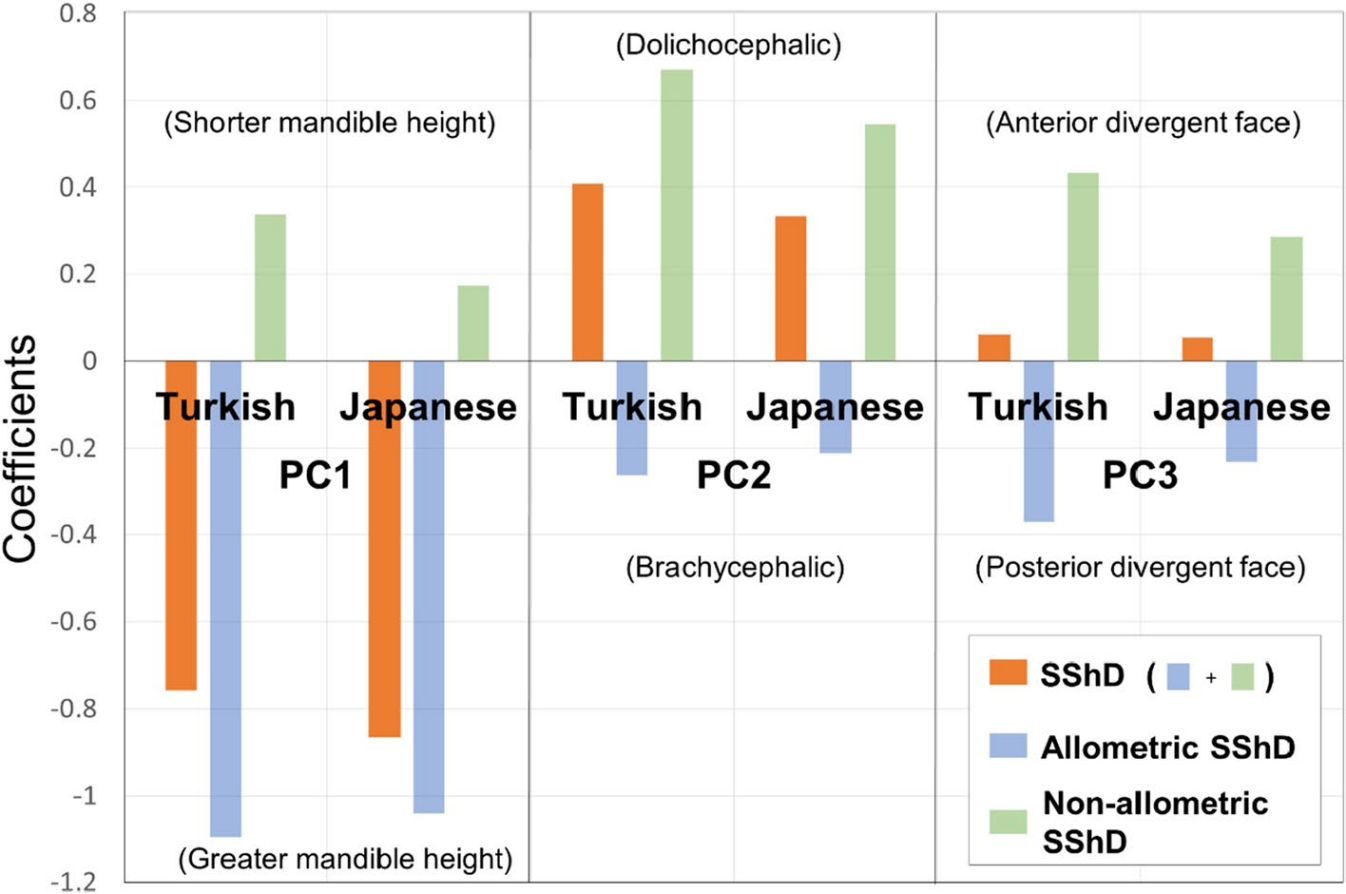
Significant coefficient values when measures of allometric and non-allometric components of sexual shape dimorphism (SShD) of individual faces were projected onto the shapes of the facial morphospace by multiple regression. *PC* principal component. All coefficient values were significant (p < 0.05).

**Figure 6:**
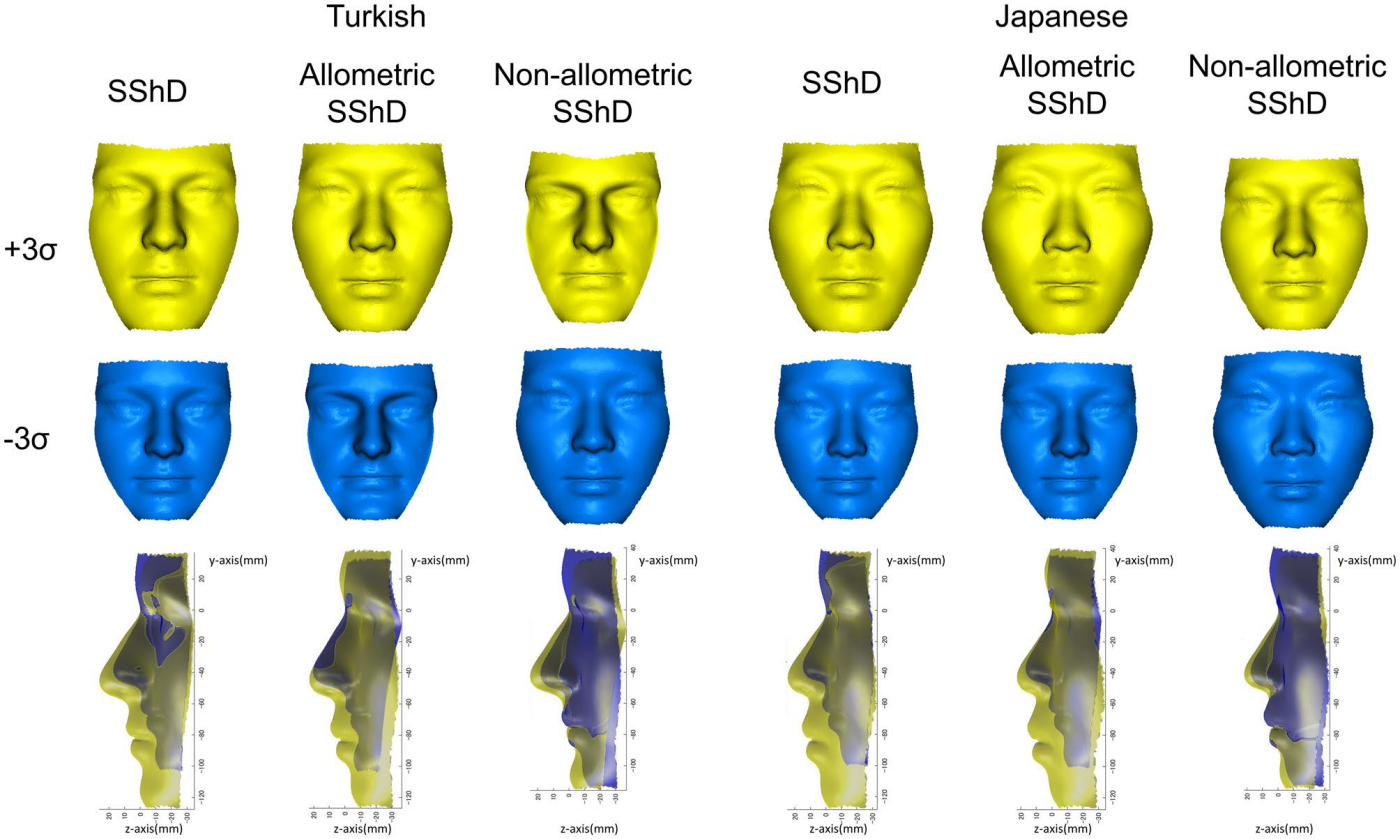
Three-dimensional visualizations of facial configuration-associated allometric and non-allometric shape differences between greater values of sexual shape dimorphism (SShD) (masculine direction; yellow) and smaller values of SShD (female direction; blue) for each population. A custom-made MATLAB based software (MATLAB 2021a, The MathWorks, Inc., Natick, MA) was used to create this figure.

A regression analysis showed that allometric SShD was mainly explained by PC 1, whereas non-allometric SShD was explained by PC 2 (Figs. 5 and 6). In both populations, males tended to have a greater anterior lower facial height than females as they grew in size and tended to have protruded noses and chins in the anterior–posterior direction, regardless of size. Facial divergency expressed as PC 3 was almost irrelevant to SShD.

### Sectional-line-and-landmark-based analyses

The results of the sectional-line-and-landmark-based analysis are shown in Supplementary Tables S1, S2, and S3, and summarized in Table 2 and Supplementary Text S1.

**Table 2:** Summary of findings in the present study that represented significant sexual dimorphism and were unique to female compared with male subjects in each respective population group.

Anatomy	Findings unique to the female subjects	Turkish	Japanese
Face height	Shorter face height due to shorter lower face height	✔	✔
	Decreased chin height	✔	
	Smaller ratio of total face height to mandible width		✔
Forehead	Flatter	✔	✔
Vertical distance between the eyes and eyebrows	Greater	✔	
Eyes	Greater eye height		✔
Face (zygomatic) width	Wider face (zygomatic) width	✔	
Cheek	Greater sagittal cheek protrusion in the posterior part of the infraorbital region and less prominence of the cheek in the parotid–masseteric region	✔	✔
Nose	Transversely and anteroposteriorly smaller in size	✔	✔
	Flatter nasal dorsum		✔
Subnasal region and upper lip vermilion	Greater retrusion of the columella base and upper lip	✔	✔
Mouth	A vertically shorter subnasale region and superiorly positioned mouth		✔
Chin	Shallow labio-mental sulcus	✔	

✔ indicates that there were significant sexual differences in facial form.

For details, see Supplementary Tables S1, S2, and S3.

Briefly, both Turkish and Japanese females had (1) a shorter lower face height, (2) a flatter forehead, (3) greater sagittal cheek protrusion in the posterior part of the infraorbital region but less prominence of the cheek in the parotid-masseteric region, and (4) an antero-posteriorly smaller nose, with a greater retrusion of the subnasal region when compared with their male counterparts.

The following phenotypic characteristics of sexual dimorphism were unique to geographical origin: Turkish females had greater vertical distance between the eyes and eyebrows, greater zygomatic width, and a shallower labio-mental sulcus; Japanese females had greater eye height, a flatter nose, a more superiorly positioned mouth, and reduced horizontal mandibular width (P < 0.01 and d > 0.8).

These results were consistent with those of the surface-based analyses.

## Discussion

In the present study, principal components that explained 66.2% of the sample’s variance showed interaction between population affinity and sex were not significant, which indicates that both populations have statistically similar expressions of sexual dimorphism. Alternatively, our detailed analysis revealed that both population affinity characteristics of facial sexual dimorphism that were common to both the Japanese and Turkish subjects, and some characteristics that were unique to each set of subjects. The presence of both similarities and differences in facial sexual dimorphism among populations described in this study were consistent with previous controversial studies regarding population affinity^9–12^. The common characteristics could have arisen from a strong selective force on fundamental function to survive as a result of natural selection, and the differing sexually dimorphic characteristics could be due to environmental adaptation under a trade-off between natural and sexual selection^22^.

Regarding the common sexual dimorphic characteristics, both the Japanese and Turkish females had a shorter face height, especially with regard to the lower face; a flatter forehead; greater sagittal cheek protrusion in the posterior part of the infraorbital region; and less prominence of the cheek in the parotid-masseteric region. Furthermore, females in both population groups had antero-posteriorly smaller noses and greater retrusion of the columella base and subnasal region.

Males showed a greater height in the lower anterior face, especially with regard to the chin, in both population groups. It should be noted that a previous cephalometric study^23^ documented temporal changes in the ratios of the anterior lower face height to the total face height in the Japanese population. Females exhibited the anterior lower face height to total face height ratio almost equal to or longer than males at 6, 8, and 10 years old. Females at 6, 10, and 14 years old had lower face height ratios that were similar to those of adults (6 years old = 54.6% and adults = 54.9%). It is after 12 years of age when males begin to have increased face height ratio^23^. The observed increase in the lower anterior face height in males can be ascribed to sexual differences in pubertal growth potential of the mandible^23^, which is prolonged in males compared with females. There are several explanations regarding why men have a greater lower anterior face height, especially in the chin after pubertal growth. From the perspective of mastication, it seems likely that the acquired basic skill for most fundamental motor performance, such as mastication and locomotion, is independent of sex^24^. A previous study^25^ documented that the smoothness or skillfulness of masticatory jaw movement in terms of minimizing the jerk cost is not sex-specific. It should, however, also be noted that some parameters, such as the amount of jaw opening and movement velocity, are sensitive to sex-specific differences in jaw size and masticatory muscle properties^25^. Adult females show longer duration and lower peak velocity in masticatory jaw movement compared with males^24^; this can be ascribed to adult males generating greater muscle force and faster muscle contraction^26^ with greater muscle volume and size of the mandible, to which the jaw-closing muscles are attached. Sex influences on maximal molar bite force and masticatory muscle thickness^17^. Thus, the anatomy and function of the masticatory muscles may contribute to explaining why males generally have greater faces, especially in the lower third.

Furthermore, the allometric decomposition findings concerning sexual shape dimorphism support the phylogenetic importance of the chewing apparatus in sexual dimorphism in males. As men require more calories than women to function^16^, it is reasonable that their greater body size tends to correlate with a greater anterior facial height for a well-developed chewing apparatus. A previous study that examined 2D allometric and non-allometric variation in the facial shape differences between men and women showed a rather weak link with allometric variation compared with non-allometric variation in most populations, including the Turkish. As our study showed that the allometric difference was greater than non-allometric differences, this is considered to be related to the sex differences in the antero-posterior direction.

From a biological perspective, sex hormones are major factors related to sexual dimorphism. In males, higher androgen serum levels at puberty exert potent osteoanabolic effects and therefore may contribute to this skeletal sexual dimorphism. Animal experiments with anabolic steroids demonstrated a clear effect on craniofacial growth, mainly as an increase in total skull length and increase in the depth of the antegonial notch^27^. Interestingly, a previous study showed that mandibular and cortical human osteoblastic cells of both sexes expressed higher androgen receptor mRNA levels and significantly more androgen binding sites per cell and exhibited significantly greater mitogenic responses to the androgen dihydrotestosterone^28^. Those results indicate that the vertically greater mandibular height in males observed in our study could be due to skeletal site-dependent expression of the androgen receptor in the mandible. Additionally, a previous study that examined facial morphology of 1-year-old boys and girls showed the existence of early sexual dimorphism, and prenatal testosterone exposure is thought to be related to sexually dimorphic facial morphology^29^. Thus, it is possible that androgens in males could contribute to facial sexual dimorphism both before and after puberty.

Previous studies on anthropoids revealed only smaller muscle strains in the supraorbital region in contrast to those in the infraorbital region or the zygomatic arch during mastication^30,31^. Animal studies^32,33^ have also revealed that circumorbital structures became greater to provide rigidity against non-masticatory forces; these studies revealed that is unlikely that masticatory muscle forces contributed to the remodeling of the supraorbital torus. On the contrary, the development of the supraorbital ridge has been viewed as an ontogenetic adaptation to masticatory forces^34^. In primates, masticatory-stress models have been examined using in vivo experimental data. Primates have significant temporalis attachments that extend to almost the midline of the frontal bones; bending of the brow-ridges is thought to be due to the mastication force pushing upward and the masseter and temporalis muscles pulling downward^35^. Few of the previous computational models, using finite element analysis of primate skulls^36^, agree with these in vivo findings. A previous study^37^ found a positive correlation between the mesio-distal crown width of the mandibular first molar and the size of the supraorbital ridge in humans. Occlusal forces exerted on the molar teeth contribute to supraorbital torus formation. Because females generate weaker muscle force and slower muscle contraction than males^26^, and exhibit decreased maximal molar bite force and masticatory muscle thickness^17^, we should not rule out the possibility of contribution of masticatory muscle forces to supraorbital ridge formation in humans. Phylogenetically, the smaller supraorbital ridge observed in the female subjects in the present study may be explained by the differences in masticatory force magnitude and its relevant jaw muscle thickness between males and females^17,30^.

In the present study, both Japanese and Turkish males showed an antero-posteriorly greater nose when the eye distances were standardized. This result is in line with those of previous studies^38–40^. Previous studies primarily hypothesized that males have evolved to have greater nasal cavity dimensions to facilitate the oxygen intake that is needed to maintain a larger body mass^37,41^. The degree of sexual dimorphism in nasal shape is considered to be potentially due to the functional integration between the nasal cavity and the respiratory system^42^.

The extent of the cheek region is defined as “superiorly to the zygomatic arch, inferiorly to the margin of the mandible, posteriorly to the ear, and anteriorly to the corner of the mouth” and is divided into four parts as topographical regions: infra-orbital, buccal, zygomatic, and parotid–masseteric regions^43^).

In the present study, in the infraorbital and buccal regions, the sagittal cheek protrusion in the posterior part of the infraorbital region was greater in the female subjects on the left side. Furthermore, lesser prominence of the cheek in the parotid–masseteric region was also observed in both Japanese and Turkish female subjects.

A lesser prominence of the cheek in the parotid–masseteric region can be explained by the smaller masseter muscles in women^17^. Thin masseter muscles lead to a lesser prominence of the cheek in the parotid–masseteric region in women.

Effects of developmental and functional interactions on morphological variability of the head through ontogeny have been discussed in previous studies^42,44^. Several studies^42,44^ have claimed that genetic signals determine the initial geometry of craniofacial anatomy, and that geometry is altered by the local mechanical environment, such as masticatory function and respiratory function, through variations in the spatio-temporal interplay of depository and resorptive activity of bone. In contrast, there is very little concrete evidence of the relationship between functional and phylogenetic development in facial configurations. In general, it is assumed that varying environmental conditions, such as climates, geographic areas, and dietary resources, require physical characteristics, including dento-facial features, which contribute to maximizing the survival probability of individuals. Hominids are now recognized as showing higher adaptability to their surrounding environment based on related morphological changes than was previously understood.

In the past, several studies have addressed 3D morphological differences between populations. For example, between Caucasians and African-Americans, the most distinct differences were observed in the forehead, alar base, and perioricular regions using 3D facial data^45^; between Caucasians and Asians, differences were observed in the malar and zygomatic areas, forehead, lips, and chin^46^. Even in the phylogenetically related populations, there were differences seen in the nasal, malar, lips, and lower facial regions between two population groups (Budapest, Hungary, and Houston, Tex)^47^; differences were also observed in the nasal width, eye distances, and facial height of two European Caucasian populations of close phylogenetic and geographic proximity (UK and Netherlands)^14^. In short, the previous studies described the facial differences between the population groups; however, limited data has been reported regarding varied facial sexual dimorphic characteristics among populations.

In the present study, four features in the Japanese and three in the Turkish were found to be exclusive sexual dimorphic characteristics. In the Japanese subjects, females had greater eye height (i.e., brighter eyes) compared with males. A medium or high upper eyelid crease is known to represent an attractive face in East Asian females, and 50% of females exhibit a minimal or absence of a double eyelid^44^. Although greater eye height is also deemed an important factor for facial attractiveness in other populations, the present results indicate that eye height is a visible facial sexual dimorphism that is more discriminatory in the Japanese subjects than the Turkish subjects.

Japanese females also showed a smaller anteroposterior protrusion of the nasal dorsum at the orbital level (i.e., a flatter nose) and a superiorly positioned mouth with a vertically shorter subnasal region. Additionally, shorter horizontal mandibular width was observed in the Japanese females. These findings indicate that Japanese females had overall smaller middle and lower facial structures than males. In a previous study that examined the 3D nasal shape and genotype in 3746 individuals, nares width was correlated with temperature and absolute humidity^48^. This result indicates that at least sexual dimorphism in nasal shape may change because of climate adaptation.

In contrast to the Japanese females, three features were found to be characteristic of the Turkish females compared with Turkish males. There was a greater vertical distance between the eyes and eyebrows, and an increased zygomatic width compared with exocanthion–exocanthion distance. These traits reflect a stout upper facial structure. Facial ontogeny research on immature hominids with a finite element model^49^ showed that bone deposition was identified over the outer aspects of the orbits, lateral nasal walls, infraorbital region, zygomatico-maxillary region, parts of the mid-clivus, including the canine jugum, and interincisal protuberance, as well as portions of the nasal sill and areas lateral to the intermaxillary suture; they inferred that these changes were related to the masticatory system^49^.

A shallower labio-mental sulcus also characterized Turkish female compared with male faces. A recent study^39^ indicated that an ontogenetic decrease in chin prominence was associated with increased vertical bending resistance and vice versa. Thus, it can be inferred that a shallow labiomental sulcus was unique to the current Turkish female participants, which indicates an adaptational response of Turkish females, who have delicately constituted jaw bones and muscles, compared with Turkish males in a dietary environment that includes tougher animal proteins compared with the Japanese dietary environment.

It is well known that Africa is the ancestral homeland of modern humans^50^. A phylogenetic tree showed the categorization of the world population into nine sub-populations based on the polymorphisms of protein genes of 1915 populations: African; North African and West Asian; European; Amerind; Arctic Northeast Asian; Northeast Asian; Southeast Asian; Pacific Islander; and New Guinean and Australian^51^. The genetic distances between Japanese (Northeast Asian) and Turkish (European) were moderately far (55% of total distance) whereas European and North African were close (7%); this indicated that Japanese and Turkish (European) had different developmental route^51^. Genetic data also provided some indication that the spread of humans into Asia was along the coast to south and south-east Asia, from where it bifurcated to the north and south^52^. Thus, our comparisons of sexual dimorphism in facial forms between Turkish and Japanese populations can explain a relatively long span of genetic drift, which is the result of population variation among individual genotypes in their probabilities of survival and/or reproduction.

Several limitations associated with the present study warrant mention. First, the Turkish population was undersampled in comparison to the Japanese population. The frontal view of our 3D Turkish data was similar to that of a previous 2D study^53^ which used a greater number of Turkish samples (n = 264); thus it could be said that our results are possibly representative of the Turkish population. However, future studies including more Turkish subjects would us to make more general conclusions. Second, our study included only two populations, so it is impossible to draw complex conclusions regarding the geographical variability of the human face. Future studies would benefit from including an even larger number of populations. Third, in the present study, we used only the centroid size of the face to examine the allometric component. The results may vary when using the height or weight. Furthermore, in the present study, we omitted color information when analyzing the data because this information was not stable among populations. In some populations, not only sexual dimorphism in facial shape but also sex differences in skin color contribute to the overall facial dimorphism. Furthermore, it has been shown that skin color is an important trait associated with facial attractiveness in populations showing high variation in skin color, especially in Africans^54,55^. This means that facial dimorphism cannot be considered only by the facial shape, and there is still a place for sexual selection that may act upon non-shape-associated facial traits. Moreover, it seems that some color traits (such as iris color) are systematically associated with the sex-specific facial shape^56,57^. Future studies using information involving sex differences in these other attributes rather than shape should be considered. Finally, although the present study does not provide a convincing explanation about whether the sexual dimorphisms, which were determined in the present study to be unique to each population group, represent consequences of natural selection for population affinity that successfully adapted to dietary environments for many generations. Therefore, although we must be cautious about the limitations of interpreting these data, the results of the present study further enhance our understanding of human sexual dimorphism expressed in the oral and facial regions.

## Conclusions

We found four facial features representing sexual dimorphism that are common to both population groups. Both Turkish and Japanese females had (1) a shorter lower face height, (2) a flatter forehead, (3) greater sagittal cheek protrusion in the posterior part of the infraorbital region but less prominence of the cheek in the parotid-masseteric region, and (4) an antero-posteriorly smaller nose, with a greater retrusion of the subnasal region when compared with their male counterparts. These results provided implications for the potential contribution of chewing apparatus in adaptation.

## Material and methods

The study was approved by the Research Ethics Committee, Osaka University Dental Hospital (project ID: H25-E37-1) and the Ethics Committee, Ankara University (project ID: 36290600/S5). All experiments were performed in accordance with relevant guidelines and regulations.

### Subjects

A total of 272 subjects, which included 72 Turkish (Turkish group; females = 36 [Turkish female subgroup]; male = 36 [Turkish male subgroup]) and 200 Japanese (females = 100 [Japanese female subgroup], males = 100 [Japanese male subgroup]) aged between 18 and 35 years, were recruited from among the students and faculty of Ankara University in Turkey and Osaka University in Japan who met the following selection criteria: no congenital facial deformities including cleft lip or palate, no facial paralysis, no noticeable scars or skin disease in the neck or dentofacial regions (or history thereof), no history of any psychiatric disorder, no subjectively or objectively discernible jaw dysfunction, a body mass index [BMI] that ranged from 18.50 to 24.99, a dental overbite that ranged from 1.0 to 5.0 mm, a dental overjet that ranged from 0.0 to 7.0 mm, and a straight soft-tissue facial profile. For the present Japanese group, we used the samples from our previous study^22^. Due to recording limitations with 3-D digital cameras, male subjects having thick beards were excluded in advance. A written informed consent form was distributed to and signed by all participants. Informed consent was approved by the Research Ethics Committee, Osaka University Dental Hospital (project ID: H25-E37-1) and the Ethics Committee, Ankara University (project ID: 36290600/S5).

### Data acquisition

The participants were asked to sit on a fixed chair with a natural head position without head support. They were then asked to assume a resting posture, which was defined as a relaxed facial posture with the lips in repose and the teeth in light contact in the habitual maximum intercuspation position. Each subject’s face was recorded once with a 3-D digital camera (3dMDcranial System, 3dMD, Atlanta, GA, USA) with a 1.5-ms capture speed and a dimensional accuracy of 0.2 mm^21^.

Each 3D facial image, scaled down to 75% of its actual size, was displayed on a 17-in LCD monitor (1701FP, Dell, Inc., Round Rock, TX, USA). The positions of 10 single and 8 paired landmarks (glabella [Gla], nasion [N], exocanthion [Ex], endocanthion [En], palpebrale superius [Ps], palpebrale inferius [Pi], porion [Po], orbitale [Or], pronasale [Prn], alar curvature point [Ac], subnasale [Sn], labiale superius [Ls], stomion [Sto], cheilion [Ch], labiale inferius [Li], submentale [Sm], pogonion [Pog], gnathion [Gn] (Supplementary Table S4, Supplementary Fig S4) were identified by visual inspection of the image and digitized using a computer mouse cursor and commercial software (Face Rugle, Medic Engineering Co., Kyoto, Japan). The zygomaticus′ [Zy′] and gonion′ [Go′] were mathematically defined as the most lateral point of the mathematically defined facial outline and the most inferior and lateral point of the mandibular facial outline, respectively, where the facial outline was defined as a series of the points with 60° angles between the surface normal vectors and Z-axis^21^. The process was repeated twice for each image, and the landmark coordinates from the two digitizations produced were averaged to yield the final landmark coordinates. A previous study^21^ that investigated the intra-observer reliability confirmed a mean absolute landmark difference of 0.32 mm (range, 0.07 to 0.52 mm) between the repeated measures (Supplementary Text S2). This result falls into the range considered reliable to highly reliable^58^.

A 3D coordinate system identical to that employed in our previous study (Supplementary Fig S4; Informed consent was obtained to publish this image in an online open-access publication.)^59^ was used in the current study. In short, the sagittal plane was defined by the exocanthions and endocanthions, and the axial plane was defined by the exocanthions, porion, and subnasale. The nasion was set as the origin.

### Analyses

The 3D soft tissue facial morphology was evaluated by the two kinds of analysis: surface- and sectional-line-and-landmark-based analyses^21,60^, which are summarized below.

**(1) Surface-based analyses**

The morphology of the facial surface was analyzed using the method documented in our previous study^21,60^.

#### Homogeneous modeling

For each facial surface, fitting of high-resolution template meshes or a generic model^60,61^ was performed using commercial software (HBM-Rugle, Medic Engineering Co., Kyoto) based on the landmarks assigned to each 3D image. This method automatically generated a homogeneous model that consisted of 6017 points (i.e., fitted mesh or semi-landmark nodes) on the wire mesh for each model with landmark anchors (i.e., Ex, En, Ps, Pi, Prn, Ac, Sn, Ls, Sto, Ch, Li, Sm, and Pog). This technique permits the extraction of relevant surface anatomy from face data while removing and/or smoothing out non-relevant data, yielding high-resolution, 3D surface data that provide enough detail to facilitate a quantitative assessment while maintaining small file sizes that are easily manipulatable and portable to a range of visualization technologies^60,61^ (Supplementary Fig S5; Informed consent was obtained to publish this image in an online open-access publication.). The arithmetic means of the coordinate values of each corresponding point on the wire mesh were computed and used to generate the averaged 3D facial images for each male and female subgroup in each population group.

The surface displacement was quantitatively evaluated in each X-, Y-, and Z-axis in two different ways. The actual displacement vectors (male to female) and significance of differences were calculated for the 6017 points on each mesh between the male and female subgroups in each population group. The calculated vectors in millimeters were visualized with color-coding. Thereafter, the arithmetic means of the coordinate values of each corresponding point on the wire mesh were statistically analyzed for significant differences between the male and female subgroups using a two-sample t-test. A significance probability map^60,61^ of the X-, Y-, and Z-values was generated to visualize these significant differences (Supplementary Fig S6).

Because previous studies revealed that masticatory muscle function likely influences mandible morphology (mainly in the vertical direction)^60^ and inter-ocular width is less affected by masticatory muscle function^62^, the eyes, which are horizontally separated paired landmarks, were considered candidates for size normalization. A previous study^36^ also showed that the right and left exocanthions were reliable points for identification. Thus, in the present study, facial size differences between individuals were standardized by normalizing the values of all surface coordinates to the distance between right and left exocanthions.

#### Sexual dimorphism of accentuated images

To quantitatively infer facial form femininity and masculinity, accentuated averaged faces, $\overline{AccA({m}_{w})}$ and $\overline{AccA({f}_{w})}$, were calculated for the male and female subgroups, respectively, to highlight site-specific sexual dimorphism^60,61^, where

$$\overline{AccA({m}_{w})}=\overline{A(m)}+w \left(\overline{A\left(m\right)} -\overline{A\left(all\right)}\right)(w=\mathrm{2,3})$$

$$\overline{AccA({f}_{w})}=\overline{A(f)}+w \left(\overline{A\left(f\right)} -\overline{A\left(all\right)}\right)(w=\mathrm{2,3})$$

and $\overline{A(m)}$, $\overline{A(f)}$, and $\overline{A\left(all\right)}$ are the arithmetic means of the coordinate values for the male subgroup, female subgroup, and the sum of both groups, respectively, and *w* is the arbitrary weight value.

#### Examination of the variance of each population and each sex, and their interactions

To examine the variance in each population and each sex, and their interactions, we first reduced dimensionality by performing PCA for the 6017 coordinates of the aforementioned surface model^63^. The significant principal components (PCs) were determined by scree plot analysis. Significant PCs were entered into a MANOVA to test for significance of the factors population affinity and sex. After MANOVA, a dendrogram was computed by applying the single linkage method to the matrix of Mahalanobis distances between subgroup means. Facial morphospace were determined by the significant PCs and used in the following process.

#### Influence of allometry on SShD

The SShD of the individual face was measured by projection of the individual facial configurations onto an axis connecting the vector between the average facial configurations of males and females in the facial morphospace using the following equation^13,64^:

$$\mathrm{SShD}(\overrightarrow{{F}_{i}}) = \frac{( \overrightarrow{{F}_{i}}\cdot \overrightarrow{{F}_{(m-f)}})}{{|\overrightarrow{{F}_{(m-f)}}|}^{2}}$$

where $\overrightarrow{{F}_{i}}$ is the vector in the facial morphospace corresponding to an individual face *i*, and $\overrightarrow{{F}_{(m-f)}}$ is the vector between male and female facial configurations (male minus female). If SShD < -1, the face is hyperfeminine, and if SShD > 1, the face is hypermasculine.

Furthermore, measures of SShD were mathematically decomposed to allometric and non-allometric components. That is, variations in SShD due to an individual’s size (allometric) and variations that were independent of size (non-allometric) were examined in the overall variation in SShD in each population group using a multivariate regression analysis. CS was used as a measure of an individual’s size. The allometric variation in SShD was calculated by regressing the shapes in the facial morphospace on CS and projecting the estimated values from this regression onto the vector of sex differences. The non-allometric component of SShD was acquired by regressing the shapes in the facial morphospace on CS and then projecting the residualized facial coordinates on the sex difference vector calculated with these residuals^13^.

To assess the differences in CS, SShD, allometric SShD and non-allometric SShD between two populations, a permutation test was conducted as a randomization test, where populations were assigned at random to facial shapes, while the gender assignment of each face and the number of men and women in each sample remained unchanged. A total of 1000 randomized samples were generated within each permutation test.

Furthermore, to understand the effect of each SShD component on the morphospace, measures of each SShD component of individual faces were projected onto the shapes the facial morphospace using multiple regression. Significant coefficient values were evaluated.

**(2) Sectional-line-and-landmark-based analyses**

To analyze the surface data in detail, five categories of curving lines were extracted from the 3D images (Supplementary Table S5). The curving lines were used to extract 142 measurements that were previously reported (see Supplementary Figs S7, S8, and S9 for definitions of those variables)^65^. In addition, 28 inter-landmark distances and 15 ratios that were previously reported^21^ were determined and employed. Therefore, 185 variables were employed in total. Facial size differences between individuals were also standardized by normalizing the values of all linear variables to the distance between right and left exocanthions.

A *t*-test was performed to determine whether the mean of each variable significantly differed between the Turkish male and female subgroups. To examine the similarity and dissimilarities of facial forms between the Japanese and Turkish groups, we included the sexual dimorphism results from a Japanese group reported in our previous study^21^. The effect size was calculated for each variable. Values greater than 0.8 for Cohen’s d were considered to have a large effect. Variables that showed significant differences and had large effects were considered biologically significant. Significance level was set to 0.01 due to a power analysis with a power of 0.8.

## Supplementary Information

Supplementary Information.

## Data Availability

The datasets generated during and/or analyzed during the current study are available from the corresponding author on reasonable request.
