# Supplementary material for: Population affinity and variation of sexual dimorphism in three-dimensional facial forms: comparisons between Turkish and Japanese populations
Source: Sci Rep. 2021 Aug 17;11:16634. doi: 10.1038/s41598-021-96029-9 (PMC8371176; doi:10.1038/s41598-021-96029-9)
Supplement: Supplementary file 1 — Supplementary Information. [file 41598_2021_96029_MOESM1_ESM.docx]

**Supporting information**

**Population affinity and variation of sexual dimorphism in three-dimensional facial forms: Comparisons between Turkish and Japanese populations**

Chihiro Tanikawa ^1,2,*^, M Okan Akcam ^3^, Hatice Gokalp ^3^, Edlira Zere ^1^, Kenji Takada ^2^

^1^ Department of Orthodontics and Dentofacial Orthopedics, Graduate School of Dentistry, Osaka University, Suita, Osaka, Japan

^2^ Center for Advanced Medical Engineering and Informatics, Osaka University, Suita, Osaka, Japan

^3^ Department of Orthodontics, School of Dentistry, Ankara University, Besevler, Ankara, Turkey

Short title: Facial Sexual Dimorphism in Turkish and Japanese

**^*^ Corresponding author:**

E-mail: ctanika@gmail.com (CT)

**Supplementary Table S1. Means, standard deviations (S.D.), and effect values of the inter-landmark distances and ratios for the Turkish and Japanese groups.**

| **Variable** | **Turkish** | | | | | | **Japanese** | | | | | | **Summary ^e)^** |
| --- | --- | --- | --- | --- | --- | --- | --- | --- | --- | --- | --- | --- | --- |
|  | **Female** | | **Male** | | **Effect value** | | **Female** | | **Male** | | **Effect value** | |  |
|  | **Mean** | **S.D.** | **Mean** | **S.D.** |  |  | **Mean** | **S.D.** | **Mean** | **S.D.** |  |  |  |
| \|Ex-Ex\| (mm) | 94.6 | 3.8 | 97.2 | 4.3 | 0.6 |  | 91.7 | 4.0 | 94.2 | 3.6 | 0.7 |  |  |
| \|Ac-Ac\| (%) ^a)^ | 36.7 | 2.7 | 39.5 | 2.1 | 1.2 | * | 40.2 | 2.2 | 42.3 | 2.3 | 0.9 | * | TJ+ |
| \|Zy-Zy\| (%) ^a)^ | 111.6 | 9.5 | 83.2 | 8.6 | 3.1 | * | 122.3 | 6.9 | 124 | 6.8 | 0.2 |  | T+ |
| \|Gla-N\| (%) ^a)^ | 19.1 | 5.4 | 14.9 | 4 | 0.9 | * | 25.7 | 6.7 | 20.8 | 3.6 | 0.9 | * | TJ+ |
| \|Gla-En\| (%) ^a)^ | 26.3 | 5.0 | 21.7 | 3.5 | 1.1 | * | 31.3 | 6.7 | 27.4 | 3.4 | 0.7 |  | T+ |
| \|Gla-Gn\| (%) Total face height ^a)^ | 138.6 | 7.5 | 145.2 | 8.6 | 0.8 | * | 148.3 | 9.6 | 152.1 | 7.4 | 0.4 |  | T+ |
| \|N-Ls\| (%) ^a)^ | 66.4 | 3.7 | 68.8 | 4.3 | 0.6 |  | 66.8 | 4.7 | 70.6 | 4.6 | 0.8 | * | J+ |
| \|N-Sto\| (%) Upper face height ^a)^ | 72.9 | 3.8 | 75 | 4.6 | 0.5 |  | 75.0 | 4.8 | 79.2 | 4.6 | 0.9 | * | J+ |
| \|N-Gn\| (%) Face height ^a)^ | 119.4 | 6.2 | 130.3 | 8.7 | 1.4 | * | 122.6 | 7.7 | 131.3 | 7.6 | 1.1 | * | TJ+ |
| \|N-Go′\| (%) ^a)^ | 26.6 | 3.4 | 30.5 | 4.4 | 1.0 | * | 28.0 | 4.5 | 29.5 | 5.4 | 0.3 |  | T+ |
| \|Ps-Pi\| (%) Eye height ^a)^ | 15.1 | 1.3 | 14.0 | 1.7 | 0.7 |  | 14.8 | 1.6 | 12.5 | 1.4 | 1.5 | * | J+ |
| \|Sn-Sto\| (%) Maxilla height ^a)^ | 16.9 | 2.7 | 17.0 | 2.4 | 0.0 |  | 20.6 | 2.6 | 22.8 | 2.7 | 0.8 | * | J+ |
| \|Sn-Gn\| (%) Lower face height ^a)^ | 61.8 | 5.2 | 70.7 | 7.1 | 1.4 | * | 65.8 | 5.8 | 72.8 | 6.8 | 1.1 | * | TJ+ |
| \|Sto-Gn\| (%) Mandible height ^a)^ | 44.9 | 4.5 | 53.7 | 7.3 | 1.4 | * | 45.2 | 5.8 | 50 | 6.2 | 0.8 |  | T+ |
| \|Gla-Sn\|/\|Sn-Gn\| Total midface-lower face height index^-a)^ | 1.22 | 0.15 | 1.04 | 0.13 | 1.3 | * | 1.23 | 0.16 | 1.07 | 0.13 | 1.1 | * | TJ+ |
| \|N-Sn\|/\|Sn-Gn\| Midface-lower face height index ^a)^ | 0.91 | 0.10 | 0.83 | 0.09 | 0.9 | * | 0.83 | 0.08 | 0.78 | 0.09 | 0.6 |  | T+ |
| \|Sn-Gn\|/\|N-Gn\| Lower face-face height index ^a)^ | 0.52 | 0.03 | 0.54 | 0.03 | 0.9 | * | 0.54 | 0.02 | 0.55 | 0.03 | 0.4 |  | T+ |
| \|Sto-Gn\|/\|N-Sto\| Mandible-upper face height index ^a)^ | 0.62 | 0.07 | 0.72 | 0.11 | 1.1 | * | 0.6 | 0.09 | 0.63 | 0.09 | 0.3 |  | T+ |
| \|Sto-Gn\|/\|Sn-Gn\| Mandible-lower face height index ^a, b)^ | 0.73 | 0.04 | 0.76 | 0.04 | 0.8 | * | 0.68 | 0.04 | 0.69 | 0.04 | 0.2 |  | T+ |
| \|Sm-Gn\|/\|Sn-Gn\| Chin-lower face height index ^a)^ | 0.45 | 0.05 | 0.51 | 0.05 | 1.2 | * | 0.43 | 0.06 | 0.44 | 0.05 | 0.2 |  | T+ |
| \|N-Gn\|/\|Zy′-Zy′\| Facialindex ^a)^ | 0.89 | 0.05 | 0.94 | 0.07 | 0.9 | * | 0.87 | 0.05 | 0.92 | 0.06 | 0.9 | * | TJ+ |
| \|N-Gn\|/\|Go′-Go′\| Face height-mandible width index ^a)^ | 1.16 | 0.08 | 1.2 | 0.12 | 0.4 |  | 1.14 | 0.09 | 1.25 | 0.13 | 1.0 | * | J+ |
| \|Sto-Gn\|/\|Go′-Go′\| Mandibular index ^a)^ | 0.43 | 0.04 | 0.49 | 0.07 | 1.0 | * | 0.42 | 0.05 | 0.48 | 0.06 | 1.1 | * | TJ+ |
| Height-to-width ratio of the outline of the supraorbital ridge ^d)^ | 0.69 | 0.25 | 0.45 | 0.25 | 0.9 | * | 0.62 | 0.24 | 0.41 | 0.18 | 1.0 | * | TJ+ |
| \|Ps-Pi\|/\|Ex-En\| Height-to-width ratio of the eye ^b)^ | 0.47 | 0.04 | 0.44 | 0.04 | 0.8 |  | 0.5 | 0.05 | 0.43 | 0.04 | 1.5 | * | J+ |

For comparison with the Japanese subjects, the effect values were calculated from the Japanese subjects that were employed in a previous study ^1^. a) Farkas and Munro, 1987^2^; b) Carre et al., 2009 ^3^ ; c)Tanikawa and Takada ^4^, 2014; d) Newly defined; e) T+ indicates significant sexual differences in the Turkish group, J+ indicates significant sexual differences in the Japanese group, and TJ+ indicates significant sexual differences in both Turkish and Japanese groups; * P<0.01 & effect value≧0.8

**Supplementary Table S2.** **Means, standard deviations (S.D.), and effect values of the Turkish and Japanese groups for the variables for the contours Ex–Ac//z, En–Ac//z, Ex–Ch//z, and Ac–Ch//z for each side of the face (See Supplementary Fig S7 for definitions).**

| **Contour** | | **Variable** | **Turkish** | | | | | | **Japanese** | | | | | | **Summary ^a)^** |
| --- | --- | --- | --- | --- | --- | --- | --- | --- | --- | --- | --- | --- | --- | --- | --- |
|  |  |  | **Female** | | **Male** | | **Effect value** | | **Female** | | **Male** | | **Effect value** | |  |
|  |  |  | **Mean** | **S.D.** | **Mean** | **S.D.** |  |  | **Mean** | **S.D.** | **Mean** | **S.D.** |  |  |  |
| Left | *Ex-Ac//z* | \|P\| (% to \|L_1_-L_2_\|) | 3.8 | 2.5 | 2.8 | 3.1 | 0.4 |  | 7.3 | 2.2 | 5.4 | 2.5 | 0.8 | * | J+ |
|  |  | ∫ (L_1_-L_2_) (% to \|L_1_-L_2_\|) | 2.2 | 1.1 | 2.4 | 1.3 | 0.2 |  | 4.4 | 1.5 | 2.8 | 1.3 | 1.1 | * | J+ |
|  | *Ex-Ch//z* | \|L_1_-L_2_\| (%) | 74.7 | 3.2 | 75.7 | 3.8 | 0.3 |  | 78.7 | 4.5 | 82.4 | 4 | 0.9 | * | J+ |
|  |  | \|P\| (% to \|L_1_-L_2_\|) | 6.7 | 4.5 | 3.4 | 4.3 | 0.8 | * | 7.8 | 3.4 | 4.2 | 2.9 | 1.1 | * | TJ+ |
|  |  | ∫ (L_1_-L_2_) (% to \|L_1_-L_2_\|) | 4.9 | 1.9 | 4.0 | 1.8 | 0.5 |  | 5.7 | 2.0 | 3.7 | 1.5 | 1.1 | * | J+ |
|  | *Ac-Ch//z* | ∠L_1_-L_2_ (°) | -9.1 | 6.0 | -6.0 | 4.5 | 0.6 |  | -6.3 | 5.9 | -1.7 | 5.2 | 0.8 | * | J+ |
|  |  | \|L_1_-L_2_\| (%) | 33.6 | 3.2 | 34.8 | 2.9 | 0.4 |  | 33.6 | 3.0 | 36.3 | 2.6 | 1.0 | * | J+ |
| Right | *Ex-Ac//z* | ∫ (L_1_-L_2_) (% to \|L_1_-L_2_\|) | 2.2 | 1.0 | 2.1 | 0.7 | 0.1 |  | 3.8 | 1.5 | 2.5 | 1.1 | 1.0 | * | J+ |
|  | *Ex-Ch//z* | ∠L_1_-L_2_ (°) | 6.8 | 3.2 | 9.6 | 4.2 | 0.8 | * | 4.8 | 3.4 | 6.5 | 3.8 | 0.5 |  | T+ |
|  |  | \|L_1_-L_2_\| (%) | 75.1 | 3.0 | 76.9 | 3.9 | 0.5 |  | 79.6 | 4.3 | 82.9 | 3.9 | 0.8 | * | J+ |
|  |  | \|P\| (% to \|L_1_-L_2_\|) | 7.6 | 4.5 | 4.6 | 5.2 | 0.6 |  | 8.0 | 3.3 | 3.7 | 2.9 | 1.4 | * | J+ |
|  |  | \|L_1_-P\| (% to \|L_1_-L_2_\|) | 49.7 | 19.5 | 42.2 | 30 | 0.3 |  | 48.6 | 14.7 | 32.9 | 22.4 | 0.8 | * | J+ |
|  |  | ∫ (L_1_-L_2_) (% to \|L_1_-L_2_\|) | 5.1 | 1.7 | 3.9 | 2.1 | 0.6 |  | 5.3 | 1.9 | 3.4 | 1.3 | 1.2 | * | J+ |
|  | *Ac-Ch//z* | ∠L_1_-L_2_ (°) | -9.3 | 5.2 | -6.1 | 4.4 | 0.7 |  | -6.6 | 5.6 | -2 | 4.5 | 0.9 | * | J+ |
|  |  | \|L_1_-L_2_\| (%) | 35.1 | 3.0 | 35.4 | 3 | 0.1 |  | 33.9 | 2.8 | 36.8 | 2.6 | 1.1 | * | J+ |
|  |  | \|L_1_-L_2_\|/L_1_^L_2_ (%) | 94.9 | 1.7 | 93.3 | 2.3 | 0.8 | * | 93.8 | 2.0 | 93 | 2.1 | 0.4 |  | T+ |

For comparisons with the Japanese subjects, the effect values were calculated from the Japanese subjects employed in a previous study ^1^.

* P<0.01 & effect value≧0.8; a) T+ indicates significant sexual differences in the Turkish group, J+ indicates significant sexual differences in the Japanese group, and TJ+ indicates significant sexual differences in both Turkish and Japanese groups

**Supplementary Table S3. Means, standard deviations (S.D.), and effect values determined for of the Turkish and Japanese groups for the variables for the contours Prn//sagittal, Gla//axial, N//axial, Or//axial, Prn//axial, Sn//axial, Ls//axial, Li//axial, and Sm//axial (see Supplementary Figs S7, S8, and S9 for definitions).**

| **Contour** | **Variable** | **Turkish** | | | | | | **Japanese** | | | | | | **Summary ^a)^** |
| --- | --- | --- | --- | --- | --- | --- | --- | --- | --- | --- | --- | --- | --- | --- |
|  |  | **Female** | | **Male** | | **Effect value** | | **Female** | | **Male** | | **Effect value** | |  |
|  |  | **Mean** | **S.D.** | **Mean** | **S.D.** |  |  | **Mean** | **S.D.** | **Mean** | **S.D.** |  |  |  |
| *Prn/sagittal* | v10 (% to \|Prn-Pog\|) | 9.0 | 2.6 | 5.3 | 3.4 | 1.2 | * | 8.6 | 2.8 | 9.2 | 4.2 | 0.2 |  |  |
| *Gla//axial* | ∠E-P-M (°, right) | 139.5 | 4.1 | 136.9 | 4.5 | 0.6 |  | 140.1 | 4.8 | 136.4 | 4.3 | 0.8 | * | J+ |
|  | \|E-E\| (%) | 135.3 | 5.1 | 139.5 | 5.0 | 0.8 | * | 142.2 | 6.7 | 143.5 | 6.2 | 0.2 |  | T+ |
| *Or//axial* | \|M\| (% to \|E-E\|) | 38.0 | 2.9 | 41.3 | 4.1 | 0.9 | * | 28.8 | 3.1 | 32 | 2.9 | 1.1 | * | TJ+ |
|  | \|N-N\| (% to \|E-E\|) | 26.7 | 2.3 | 27.9 | 2.5 | 0.5 |  | 21.7 | 3.3 | 24.9 | 2.6 | 1.1 | * | J+ |
|  | ∠N-M (°, left) | 41.6 | 4.4 | 38.4 | 3.8 | 0.8 | * | 53.7 | 6.6 | 48 | 3.8 | 1.1 | * | TJ+ |
|  | \|P\| (% to \|E-E\|, left) | 8.7 | 1.0 | 7.8 | 1.2 | 0.8 | * | 11.4 | 1.3 | 10.2 | 1.4 | 0.9 | * | TJ+ |
|  | ∠E-P-N (°, right) | -69.8 | 2.5 | -67.2 | 2.7 | 1.0 | * | 106.7 | 2.4 | 108.1 | 3.0 | 0.5 |  | T+ |
|  | ∠N-M (°, right) | 40.3 | 4.0 | 37.6 | 3.8 | 0.7 |  | 54.1 | 6.9 | 47.5 | 4.1 | 1.2 | * | J+ |
|  | \|P\| (% to \|E-E\|, right) | 9.1 | 0.9 | 7.8 | 1.2 | 1.2 | * | 11.5 | 1.5 | 10.3 | 1.5 | 0.8 | * | TJ+ |
|  | \|E-E\| (%) | 127.5 | 5.3 | 131.7 | 4.6 | 0.8 | * | 135 | 7 | 137.1 | 7.1 | 0.3 |  | T+ |
| *Prn//axial* | \|M\| (% to \|E-E\|) | 43.4 | 3.0 | 47.1 | 3.7 | 1.1 | * | 35.4 | 3 | 39.2 | 2.9 | 1.3 | * | TJ+ |
|  | ∠E-P-N (°, right) | -72.8 | 2.9 | -66.3 | 6.8 | 1.3 | * | 104 | 3.8 | 106.6 | 4.5 | 0.6 |  | T+ |
|  | ∠N-M (°, right) | 32.3 | 2.9 | 35.4 | 11.6 | 0.4 |  | 41.6 | 4.8 | 38.3 | 3.7 | 0.8 | * | J+ |
|  | \|P\| (% to \|E-E\|, right) | 9.5 | 1.2 | 7.1 | 2.3 | 1.3 | * | 11.3 | 1.5 | 10.2 | 1.9 | 0.6 |  | T+ |
|  | \|E-E\| (%) | 121.6 | 6.1 | 127.3 | 5.3 | 1.0 | * | 129.9 | 7.2 | 132.6 | 7.6 | 0.4 |  | T+ |
| *Sn//axial* | \|M\| (% to \|E-E\|) | 15.6 | 1.5 | 17.7 | 1.9 | 1.2 | * | 13.9 | 1.6 | 15.7 | 1.7 | 1.1 | * | TJ+ |
|  | \|E-E\| (%) | 117.8 | 6.5 | 124.4 | 6.4 | 1.0 | * | 125.2 | 7.5 | 126.7 | 8.8 | 0.2 |  | T+ |
| *Ls//axial* | \|M\| (% to \|E-E\|) | 17.1 | 1.6 | 19 | 1.7 | 1.1 | * | 16.7 | 1.6 | 18.6 | 1.8 | 1.1 | * | TJ+ |
|  | \|P\| (% to \|E-E\|, left) | 7.1 | 1.6 | 8.9 | 2.4 | 0.9 | * | 7.6 | 1.5 | 8.8 | 2.3 | 0.6 |  | T+ |
|  | \|P\| (% to \|E-E\|, right) | 7.2 | 1.3 | 8.7 | 2.3 | 0.8 | * | 7.5 | 1.4 | 9.2 | 2.5 | 0.8 | * | TJ+ |
| *Li//axial* | \|E-E\| (%) | 109 | 7.1 | 115.1 | 7.7 | 0.8 | * | 112.7 | 8.4 | 111.3 | 11.2 | 0.1 |  | T+ |
|  | \|M\| (% to \|E-E\|) | 19 | 1.7 | 19.2 | 1.7 | 0.1 |  | 18.7 | 1.6 | 20.2 | 2.1 | 0.8 | * | J+ |
|  | \|P\| (% to \|E-E\|, left) | 8.7 | 1.7 | 9.7 | 2.3 | 0.5 |  | 8.4 | 1.7 | 10.4 | 2.7 | 0.9 | * | J+ |
|  | \|P\| (% to \|E-E\|, right) | 8.1 | 1.8 | 9.2 | 2.2 | 0.5 |  | 7.8 | 1.7 | 9.9 | 3.0 | 0.9 | * | J+ |
| *Sm//axial* | \|P\| (% to \|E-E\|, left) | 13.3 | 2.5 | 13.9 | 3.3 | 0.2 |  | 12.4 | 2.5 | 14.7 | 3.1 | 0.8 | * | J+ |
|  | ∠E-P-M (°, right) | 148.8 | 3.4 | 145.9 | 3.2 | 0.9 | * | 149.7 | 3.9 | 146.3 | 3.9 | 0.9 | * | TJ+ |
|  | \|P\| (% to \|E-E\|, right) | 11.6 | 2.7 | 12.0 | 2.9 | 0.2 |  | 10.6 | 2.1 | 13.3 | 3.2 | 1.0 | * | J+ |

For comparisons with the Japanese group, the effect values of the Japanese group were calculated based on data from a previous study^1^. * P<0.01 & effect value≧0.8; a) T+ indicates significant sexual differences in the Turkish group, J+ indicates significant sexual differences in the Japanese group, and TJ+ indicates significant sexual differences in both Turkish and Japanese groups

**Supplementary Table S4.** Definitions of the soft tissue landmarks on the facial 3-D images

| Landmark |  | Definition | Single | Paired |
| --- | --- | --- | --- | --- |
| Glabella | Gla | The most prominent midpoint between the eyebrows (Mathematically defined) | ✓ |  |
| Nasion | N | The deepest point on the nasofrontal region in a sagittal plane | ✓ |  |
| Exocanthion | Ex | The point at the outer commissure of the eye fissure |  | ✓ |
| Endocanthion | En | The point at the inner commissure of the eye fissure |  | ✓ |
| Palpebrale superius | Ps | The highest point on the middle portion of the free margin of each upper eyelid |  | ✓ |
| Palpebrale inferius | Pi | The lowest point on the middle portion of the free margin of each lower eyelid |  | ✓ |
| Porion | Po | The most superior point on each ear canal |  | ✓ |
| Orbitale | Or | The lowest point on the margin of the orbitale area |  | ✓ |
| Zygomaticus | Zy | The most prominent point on the zygomatic area from the 45° oblique view (Mathematically defined) |  | ✓ |
| Pronasale | Prn | The most protruding point of the apex nasi in a sagittal plane | ✓ |  |
| Alar curvature point | Ac | The most posterolateral point in the curved base line of the alar, indicating the facial insertion of the base of the nasal wing base onto the curvature of the base |  | ✓ |
| Subnasale | Sn | The midpoint of the base of the columella where the lower border of the nasal septum meets the surface of the upper lip | ✓ |  |
| Labiale superius | Ls | The outermost point on the mucocutaneous border of the upper lip in the mid-sagittal plane | ✓ |  |
| Stomion | Sto | The point between the lowermost point on the vermillion of the upper lip and the uppermost point on the vermillion of the lower lip in the mid-sagittal plane | ✓ |  |
| Cheilion | Ch | The outer corner of the mouth where the outer edges of the upper and lower vermilions meet |  | ✓ |
| Labiale inferius | Li | The outermost point on the mucocutaneous border of the lower lip in the mid-sagittal plane | ✓ |  |
| Submentale | Sm | The deepest point of the midline concavity between the lower lip and chin in the mid-sagittal plane | ✓ |  |
| Pogonion | Pog | The most prominent point of the chin in the mid-sagittal plane | ✓ |  |
| Gnathion | Gn | The most anterior inferior point of the chin in the mid-sagittal plane (Mathematically defined; see^1^) | ✓ |  |
| Zygomaticus′ | Zy′ | The most lateral point on the facial outline (Mathematically defined; see^1^) |  | ✓ |
| Gonion′ | Go′ | The most inferior and lateral point on the external angle of the mandible (Mathematically defined; see ^1^) |  | ✓ |

**Supplementary Table S5. Five categories of curving lines.**

| **Category** | **Variable (Contour)** | **Landmark 1** | **Landmark 2** |
| --- | --- | --- | --- |
|  |  | **(L_1_)** | **(L_2_)** |
| Inter-landmark contour parallel to the Z-axis | *Ex-Ac//z* | *Ex* | *Ac* |
|  | *En-Ac//z* | *En* | *Ac* |
|  | *Ex-Ch//z* | *Ex* | *Ch* |
|  | *Ac-Ch//z* | *Ac* | *Ch* |
| Sagittal section (parallel to the Z-Y plane) | *N//sagittal* | *N* | *Sn* |
|  | *Prn//sagittal* | *Prn* | *Pog* |
| Axial section (parallel to the Z-X plane) | *Gla//axial* | *Gla* | N/A |
|  | *N//axial* | *N* | N/A |
|  | *Sn//axial* | *Sn* | N/A |
|  | *Ls//axial* | *Ls* | N/A |
|  | *Li//axial* | *Li* | N/A |
|  | *Sm//axial* | *Sm* | N/A |
|  | *Or//axial* | *Or* | N/A |
|  | *Prn//axial* | *Prn* | N/A |
| Facial outline | *Facial outline* | N/A | N/A |
| Supraorbital ridge outline | *Supraorbital ridge outline* | N/A | N/A |

For definitions, Please see ^1^. N/A: not applicable.

**
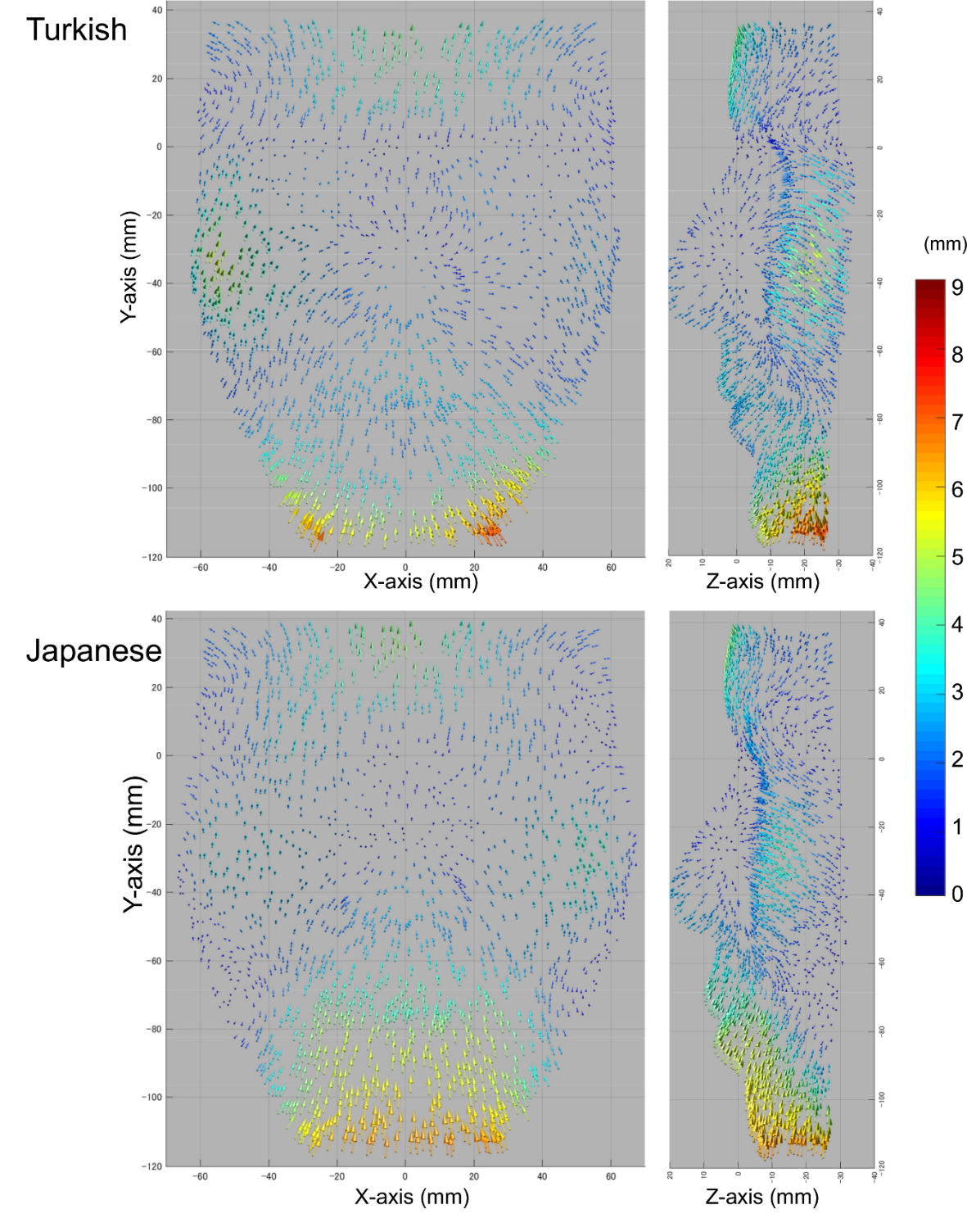
Supplementary Fig S1. Vectors from the average mesh points of the male group (arrow base) to those of the female group (arrow tip).** Greater scalar values are indicated by red, and smaller values are indicated by blue. Left, frontal view; right, lateral view; top, Turkish; bottom, Japanese.


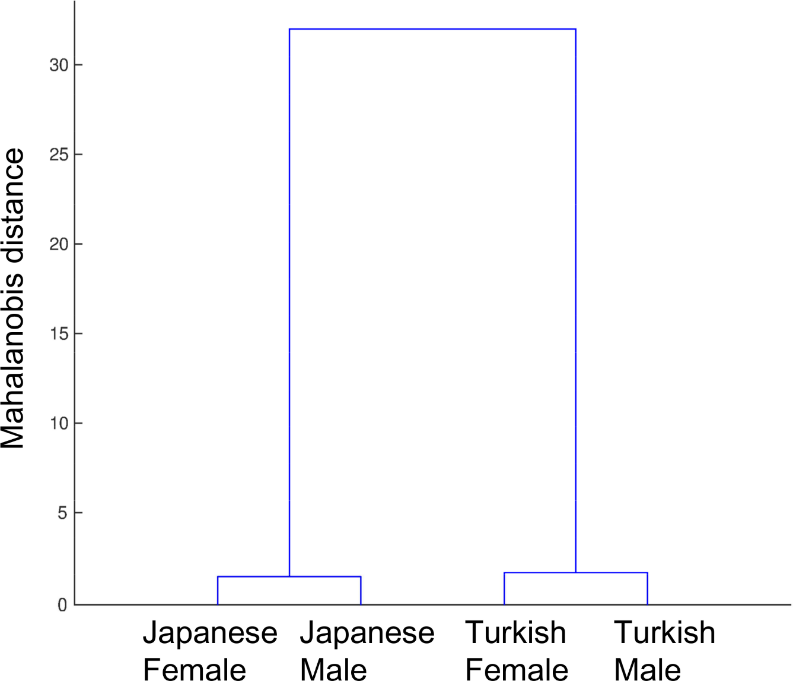


**Supplementary Fig S2.** **Dendrogram produced by applying the single linkage method to the matrix of Mahalanobis distances between subgroup means.**


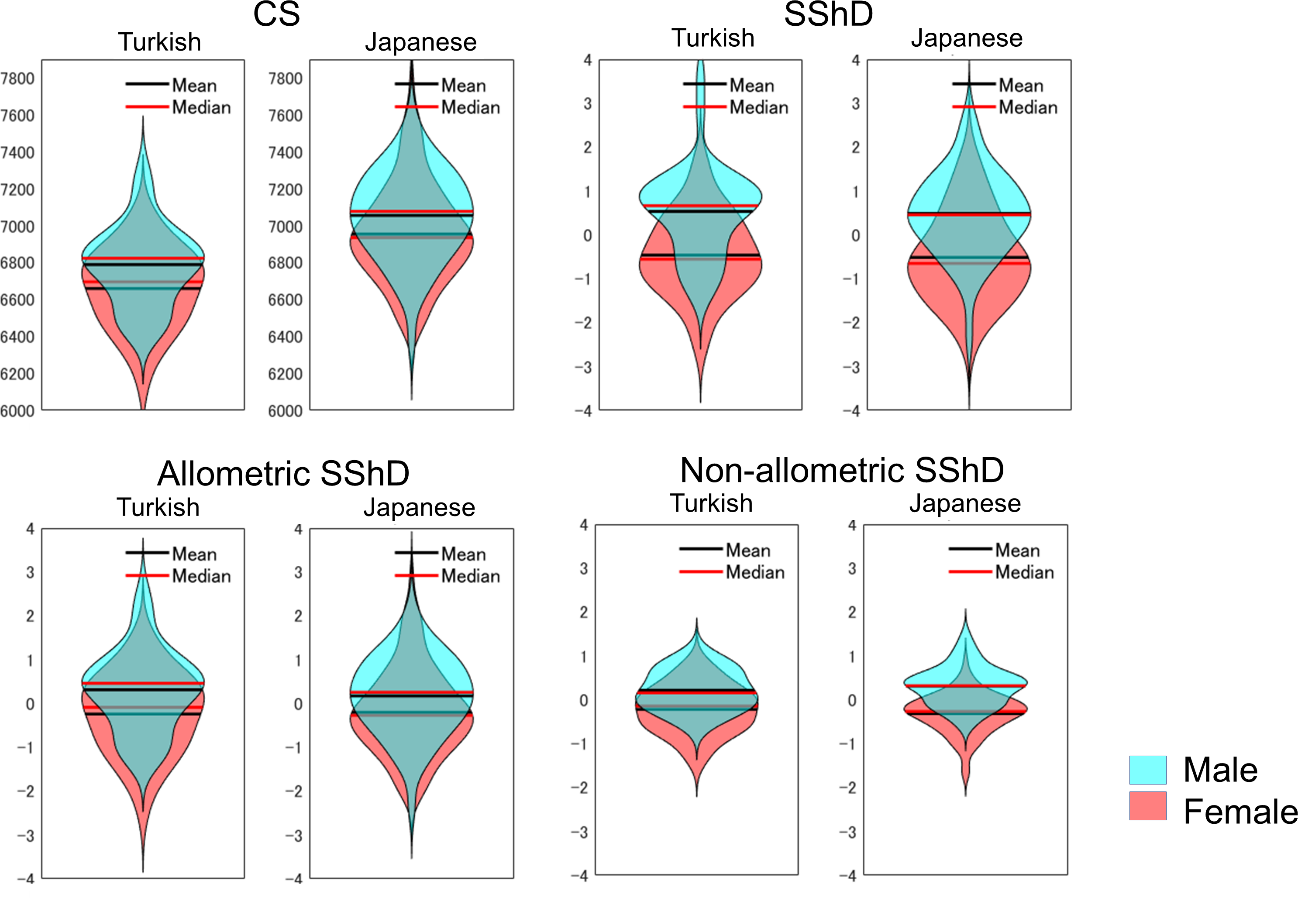
 **Supplementary Fig S3.** Violin plots showing the range and variation in centroid size (CS) and sexual shape dimorphism (SShD; overall, allometric, and non-allometric) across two populations. Light blue indicates males, and red indicates females.


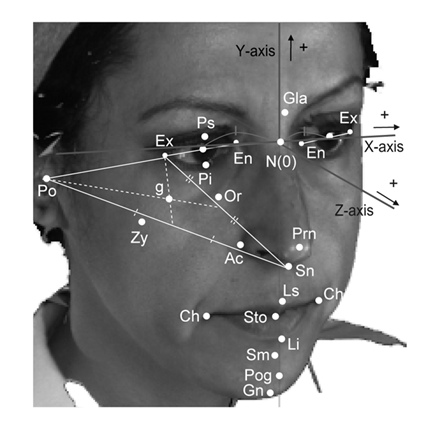


**Supplementary Fig S4.** **Coordinate system and landmarks employed in the present study.** See Supplementary Table S4 for definitions of the landmarks. See Tanikawa et al., 2016 ^1^ for details. Informed consent was obtained to publish this image in an online open-access publication.

S
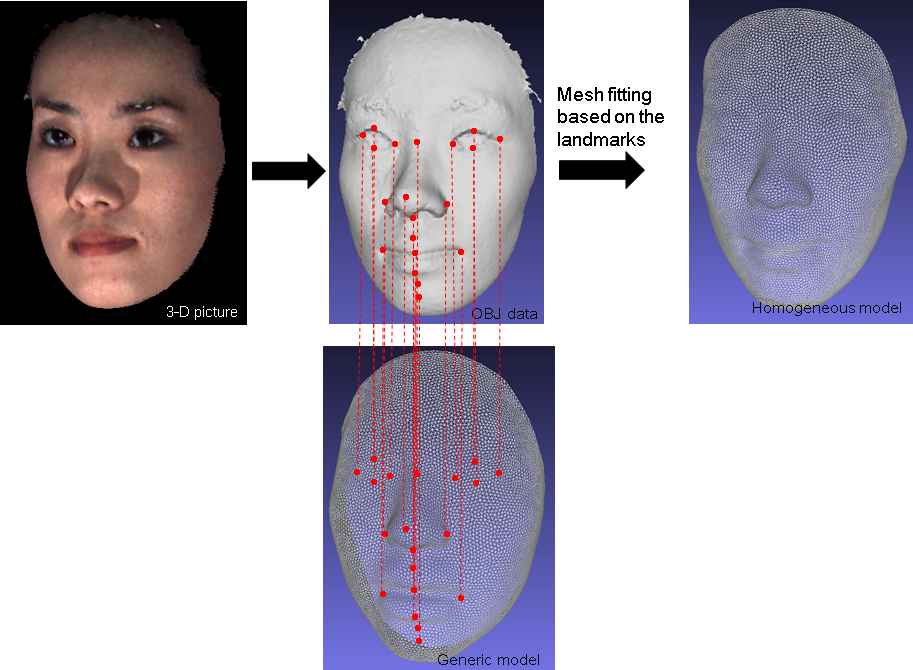


**Supplementary Fig S5. Schematic illustration of the wire mesh fitting and the point cloud of the face that were examined.** For each facial surface (top left), fitting of high-resolution template meshes or a generic model (bottom center) was performed using commercial software (HBM-Rugle, Medic Engineering Co., Kyoto) based on the landmarks assigned to each 3D image (top center). This method automatically generated a homogeneous model (top right) that consisted of 6,017 points (i.e., fitted mesh or semi-landmark nodes) on the wire mesh for each model with landmark anchors. The semi-landmark nodes on the wire mesh of the homogeneous model generated for each face were used for further calculation, as in Supplementary Fig S6. Informed consent was obtained to publish this image in an online open-access publication.


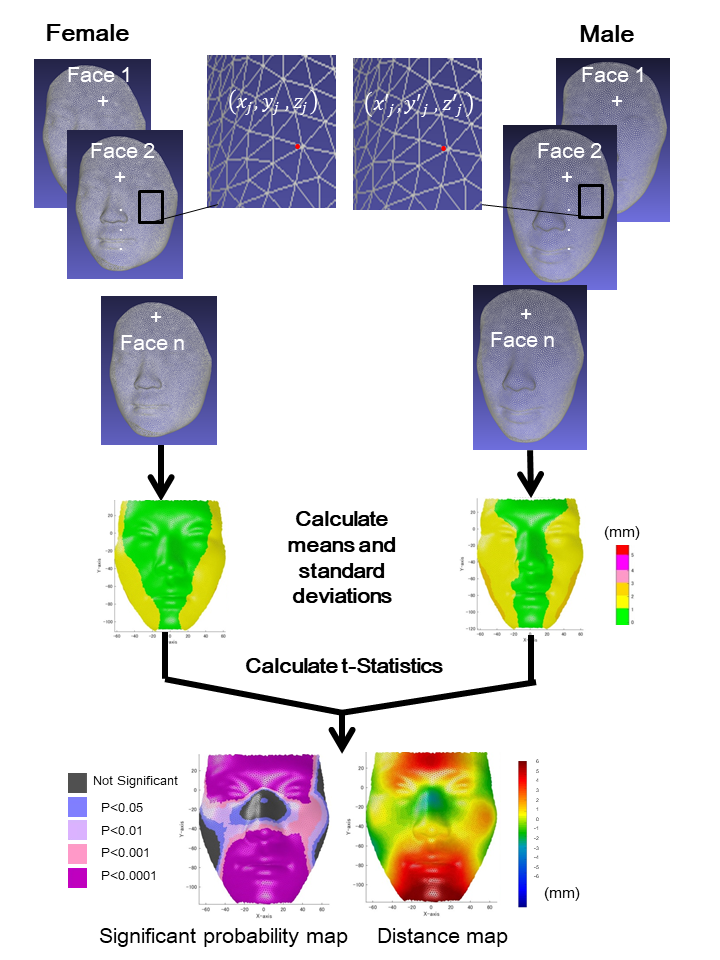


**Supplementary Fig S6.** **Schematic illustration of computation for the significant probability map and distance map.** The semi-landmark nodes on the wire mesh of the homogeneous model generated for each face (top left and right) were used for calculation. First, for each node, the mean and variance for each female and male group were calculated. Second, the t-statistics between female and male groups were represented as a color map. Colors at each node represent the averaged facial forms for each group. A custom-made MATLAB based software (MATLAB 2021a, The MathWorks, Inc., Natick, MA) was used to create this figure.


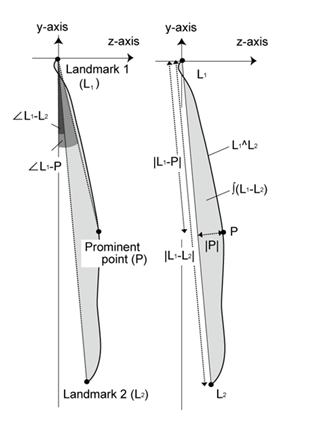


**Supplementary Fig S7. Schematic diagram that illustrates the measurements for the contours Ex–Ac//z, En–Ac//z, Ex–Ch//z, and Ac–Ch//z.** See Tanikawa et al., 2016 for details. Landmark 1 (L1; see Supplementary Table S5) was defined as the origin of the system. The z-axis was defined as the line that passes through the origin and is parallel to ground Z-axis in Supplementary Fig S4. The y-axis was defined as the line perpendicular to the z-axis that passes through the origin. ∠L1–L2 designates the angle formed by the line that connects L1 and L2, and the y-axis (protrusion of L2 relative to L1); |L1–L2| designates the distance between L1 and L2; |P| designates the distance between the line L1–L2 and the most prominent point (P) on the curving line L1–L2; |L1–P| designates the distance between L1 and the base of the most prominent point along the line L1–L2; ∫(L1–L2) designates the area enclosed by the line L1–L2 and the contour; ∠L1–P designates the angle formed by the line that connects L1 and the most prominent point, and the y-axis; and L1^L2 designates the length of the contour along its curvature.


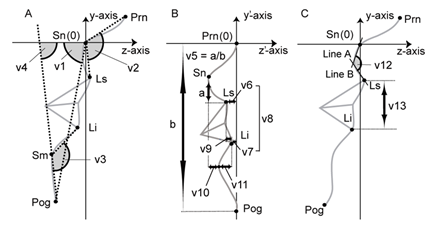


**Supplementary Fig S8. Measurements for the contour Prn//sagittal (i.e., naso–lip–chin profile^5,6^).** (A) Schematic diagram that illustrates vector elements v1, v2, v3, and v4. Sn was defined as the origin of the system. The z-axis was defined as the line that passes through the origin and is parallel to ground Z-axis in Supplementary Fig S4. The y-axis was defined as the line perpendicular to the z-axis that passes through the origin. The gray line denotes the contour Prn//sagittal. (B) Schematic diagram that illustrates the definitions of vector elements v5, v6, v7, v8, v9, v10, and v11. Prn was defined as the origin, the y′-axis as the line that connects Prn and Pog, and the z′-axis as the line perpendicular to the y′-axis that passes through the origin. (C) Schematic diagram that illustrates the definitions of vector elements v12 and v13. Sn was defined as the origin, the z-axis as the line through the origin and parallel to ground Z-axis in Supplementary Fig S4, and the y-axis as the line through the origin perpendicular to the z-axis. Lines A and B are first-order polynomial approximations generated from the extracted contour data (for line A, the data were extracted from Sn to the midpoint of Sn and Ls; for line B, the data were extracted from Ls to the midpoint of Sn and Ls). v1 designates the angle formed by the Sn–Pog line and the z-axis (which indicates the degree of protrusion of the mandible, with a large value indicating greater protrusion); v2 designates the angle formed by the Prn–Sn and Sn–Ls lines (which indicates the naso-labial angle); v3 designates the angle formed by the Li–Sm and Sm–Pog lines, the labio-mental angle; v4 designates the angle formed by the Sm–Pog line and z-axis (which indicates the degree of prominence of the chin, with a small value indicating greater prominence); v5 designates the value of [the difference between the y′-coordinate values of Sn and Ls]/[the difference between the y′-coordinate values of Prn and Pog] (which indicates the vertical length of the subnasal region); v6 designates the z′ coordinate value of Ls, the sagittal position of the upper lip vermilion, with a positive value indicating protrusion of the upper lip relative to the line that connects Prn and Pog; v7 designates the z′-coordinate value of Li, with a positive value indicating protrusion of the lower lip relative to the line that connects Prn and Pog; v8 designates the value of (v6+v7) (which indicates the sagittal position of the upper and lower lip vermilions, with a positive value indicating bilabially protruding lip vermilions); v9 designates the value of (v7–v6) (which indicates the sagittal relationship between the upper and lower lips, with a positive value indicating a protuberant lower lip relative to its upper counterpart); v10 designates the difference between the z′-coordinate values of Sn and Sm (which indicates the degree of protrusion of the labio-mental sulcus); v11 designates the difference between the z′-coordinate values of Sm and Li (which indicates the depth of the labio-mental sulcus); v12 designates the angle formed by the approximated lines A and B, where line A was defined as an approximated line between Sn and the midpoint of Sn and Ls, and line B as an approximated line between the midpoint of Sn and Ls [which indicates the subnasal (Sn–Ls line) form, with a small value that indicates a backward-curving subnasal form]; and v13 designates the difference between the y-coordinate values of Ls and Li (which indicates the vertical thickness of the lip vermilions). All linear variables were normalized to the difference between the y-coordinate values of Prn and Pog.


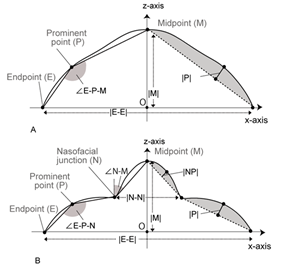


**Supplementary Fig S9. Schematic diagrams that illustrate the measurements of the contours.** (A) The contours Gla//axial, N//axial, Sn//axial, Ls//axial, Li//axial, and Sm//axial. See Tanikawa et al., 2016 for details. The endpoints (E) were defined as the two endpoints of the surface contour, the midpoint (M) as the mid-sagittal point on the surface contour, and the prominent point (P) as the most prominent point on the skin surface contour between the endpoint and midpoint. The midpoint of the endpoints was defined as the origin (O) of the system. The x-axis was defined as the line that passes through the origin and is parallel to ground X-axis in Supplementary Fig S4. The z-axis was defined as the line perpendicular to the x-axis that passes through the origin. The variable |E–E| designates the distance between left E and right E; |M| designates the distance between M and a line that connects the left and right endpoints (which indicates mid-sagittal prominence); ∠E–P–M designates the angle formed by E, P, and M (a small value indicates greater cheek prominence); and |P| designates the distance between P and the line that connects E and M (which indicates cheek prominence). (B) Or//axial and Prn//axial contours. The endpoints were defined as the two endpoints of the surface contour; the midpoint as the mid-sagittal point on the surface contour; the nasofacial junction (N) as the nasofacial junction point on the surface contour; the prominent point as the most prominent point on the surface contour between the endpoint and nasofacial junction; and the nasal prominent point (NP) as the most prominent point on the surface contour between the nasofacial junction and the midpoint. The midpoint of the endpoints was defined as the origin of the system. The x-axis was defined as the line that passes through the origin and is parallel to ground X-axis in Supplementary Fig S4. The z-axis was defined as the line perpendicular to the x-axis that passes through the origin. |N–N| designates the distance between the two nasofacial junction points (which indicates the width of the nose at the level of Or or Prn); ∠E–P–N designates the angle formed by E, P, and N; |P|, the distance between P and the line that connects E and N (which indicates cheek prominence); ∠N–M designates the angle formed by the z-axis and the line that connects N and M (which indicates the inclination of the nasal sidewall); and |NP| designates the distance between NP and the line that connects N and M (which indicates the nasal–dorsum prominence for the Or//axial contour and nasal–alar prominence for the Prn//axial contour).

**Supplementary Text S1. Detailed results of sectional-line-and-landmark-based analyses**

We categorized these variables into three categories, i.e., (1) common characteristics between two ethnic groups, (2) phenotypic characteristics unique to Turkish, and (3) that to Japanese. No variable showed opposite results regarding sexual differences between the Japanese and Turkish subject groups.

***Category 1. Sexual dimorphism commonly seen in both Japanese and Turkish subjects.*** Sixty-eight out of the 185 variables significantly differed (P<0.01) between the male and female subgroups, and Cohen’s d >0.8 was found in both the Japanese and Turkish subject groups (Supplementary Tables S1, S2, S3). Major differences were determined in the facial proportion, forehead, nose, upper lip, and cheeks.

***Facial proportion.*** Overall, the female subjects had shorter face height compared with the male subjects due to the enhanced lower face height (|Sn–Gn|, |Gla–Sn|/|Sn–Gn|).

The lower face height to mandibular width was also smaller in the female than male subgroups (|Sto–Gn|/|Go′–Go′|).

***Forehead.*** The forehead was flatter in the female subgroups and more sloped from the eyebrows to the root of the nose in the male subgroups (|Gla–N|). Less vertical and/or greater horizontal protrusion of the forehead in the male subjects compared with the female subjects was also observed as a sexual dimorphism commonly seen in both the Japanese and Turkish subjects. Additionally, in contrast to the flat and round shapes of the females’ foreheads, the males showed prominent supraorbital ridges, which can be observed as a distinct broader extension of the contours above the eyes.

**Nose.** The nose was transversely and antero-posteriorly smaller in size in the female subjects (|Ac–Ac|, Or//axial).

***Upper lip.*** The female subgroups showed a greater retrusion of the upper lip vermilion in the female subjects (Ls//axial). The averaged female faces with enhanced femininity showed grooves that ran from each side of the nose to the corners of the mouth below the cheeks; this is associated with prominent upper lips and cheeks. In contrast, the contour maps of the accentuated averaged male faces with enhanced masculinity showed a transversely wider and flatter shape of the upper lips.

***Cheeks.*** The sagittal cheek protrusion in the posterior part of the infraorbital region and in the parotid–masseteric region, was greater in the female subjects (Ex-Ch//z, Or//axial, Ls//axial).

**Category 2. Phenotypic characteristics of sexual dimorphism unique to the Turkish subjects (Turkish positive/Japanese negative)**

***Distance between the eyes and eyebrows.*** The Turkish female subgroup showed a greater vertical distance between the eyes and eyebrows compared with the Turkish male subgroup (|Gla–En|). This may be explained by the sloped forehead and the prominent supraorbital ridges found in the Turkish male subjects.

***Chin.*** Turkish females had decreased chin height compared with Turkish males, whereas the Japanese subjects did not show any significant sexual differences with regard to those indices (|N–Go′|, |N–Sn|/|Sn–Gn|, |Sn–Gn|/|N–Gn|, Sto–Gn|/|N–Sto|, |Sto–Gn|/|Sn–Gn|, |Sm–Gn|/|Sn–Gn|). The antero-posterior difference in distance between the subnasale and submentale (v10 in Supplementary Fig S8, which indicates the degree of protrusion of the labio-mental sulcus) was greater in the female than male subgroup for the Turkish subjects. This means that Turkish females had an anterior-posteriorly shallow labio-mental sulcus.

**Category 3. Phenotypic characteristics of sexual dimorphism unique to the Japanese subjects (Japanese positive/Turkish negative)**

***Mandible width.*** Japanese female subjects exhibited a smaller ratio of face height to mandibular width compared with the Japanese male subjects (|N–Gn|/|Go′–Go′|).

***Eyes.*** The Japanese female subgroup showed greater eye height than the male subgroup (|Ps–Pi|, Ps–Pi|/|Ex–En|). This is consistent with the contour maps of the accentuated averaged faces; the averaged female faces with enhanced femininity showed vertically larger eye fissures, whereas the subjects in the male group had vertically smaller eyes.

***Nose.*** The nasal dorsum was flatter at the orbital level in the Japanese female subgroup (Or//axial).

***Vertical position of the mouth.*** The Japanese female subgroup had a superiorly positioned mouth compared with the Japanese male subgroup (|N–Ls| and |N–Sto|). Furthermore, the subnasal region was vertically shorter in the female than male subgroup (|Sn–Sto|, Ex–Ch//z, Ac–Ch//z).

**Supplementary Text S2. Reliability**

We conducted both surface- and sectional-line-and-landmark-based analyses, based on the landmarks assigned to each acquired 3D image. Our methods automatically generated a homogeneous model and 185 sectional-line-and-landmark-based variables. Thus, we regard the reliability of these two methods entirely as a consequence of the identification of landmarks. The intra-observer reliability of these landmarks was confirmed in a previous study^1^. Briefly, to determine the intra-observer reliability of landmark identification, 3D facial images of 15 participants were randomly selected from among the subjects of the previous study, and the coordinate values of the landmarks (nasion, exocanthion, endocanthion, palpebrale superius, palpebrale inferius, porion, orbitale, pronasale, alar curvature, subnasale, labiale superius, stomion, cheilion, labiale inferius, submentale, pogonion) were determined. The digitization process was then repeated 1 week later, and the two sets of results were compared by using a paired t-test and correlation analyses. The investigation of intra-observer reliability showed a mean absolute difference of 0.13 mm (range, 0.00 mm [pronasale] to 0.52 mm [pogonion]; P > 0.01) and 0.04 mm (range, 0.00 mm [pronasale] to 0.17 mm [submentale]; P > 0.01) for the two examiners, respectively. Correlations between the two sets of results ranged from 0.97 to 1.00 (P < 0.01), across the landmarks.

**References**

1 Tanikawa, C., Zere, E. & Takada, K. Sexual dimorphism in the facial morphology of adult humans: A three-dimensional analysis. *Homo* **67**, 23-49, doi:10.1016/j.jchb.2015.10.001 (2016).

2 Farkas, L. G. *Anthropometry of the head and face*. (Raven Press, 1994).

3 Carre, J. M., McCormick, C. M. & Mondloch, C. J. Facial structure is a reliable cue of aggressive behavior. *Psychol Sci* **20**, 1194-1198, doi:DOI 10.1111/j.1467-9280.2009.02423.x (2009).

4 Tanikawa, C. & Takada, K. Objective classification of nose-lip-chin profiles and their relation to dentoskeletal traits. *Orthodontics & Craniofacial Research* **17**, 226-238, doi:10.1111/ocr.12047 (2014).

5 Tanikawa, C., Kakiuchi, Y., Yagi, M., Miyata, K. & Takada, K. Knowledge-dependent pattern classification of human nasal profiles. *Angle Orthod* **77**, 821-830, doi:10.2319/061806-247.1 (2007).

6 Tanikawa, C., Nakamura, K., Yagi, M. & Takada, K. Lip vermilion profile patterns and corresponding dentoskeletal forms in female adults. *Angle Orthod* **79**, 849-858, doi:10.2319/103008-552.1 (2009).
